# Controlled Self-assembly of Stem Cell Aggregates Instructs Pluripotency and Lineage Bias

**DOI:** 10.1038/s41598-017-14325-9

**Published:** 2017-10-25

**Authors:** Angela W. Xie, Bernard Y. K. Binder, Andrew S. Khalil, Samantha K. Schmitt, Hunter J. Johnson, Nicholas A. Zacharias, William L. Murphy

**Affiliations:** 1 Department of Biomedical Engineering, University of Wisconsin-Madison, Madison, WI 53705 United States; 2 Department of Surgery, University of Wisconsin-Madison, Madison, WI 53705 United States; 3 Department of Materials Science and Engineering, University of Wisconsin-Madison, Madison, WI 53705 United States; 4 Department of Orthopedics and Rehabilitation, University of Wisconsin-Madison, Madison, WI 53705 United States

## Abstract

Stem cell-derived organoids and other 3D microtissues offer enormous potential as models for drug screening, disease modeling, and regenerative medicine. Formation of stem/progenitor cell aggregates is common in biomanufacturing processes and critical to many organoid approaches. However, reproducibility of current protocols is limited by reliance on poorly controlled processes (e.g., spontaneous aggregation). Little is known about the effects of aggregation parameters on cell behavior, which may have implications for the production of cell aggregates and organoids. Here we introduce a bioengineered platform of labile substrate arrays that enable simple, scalable generation of cell aggregates via a controllable 2D-to-3D “self-assembly”. As a proof-of-concept, we show that labile substrates generate size- and shape-controlled embryoid bodies (EBs) and can be easily modified to control EB self-assembly kinetics. We show that aggregation method instructs EB lineage bias, with faster aggregation promoting pluripotency loss and ectoderm, and slower aggregation favoring mesoderm and endoderm. We also find that aggregation kinetics of EBs markedly influence EB structure, with slower kinetics resulting in increased EB porosity and growth factor signaling. Our findings suggest that controlling internal structure of cell aggregates by modifying aggregation kinetics is a potential strategy for improving 3D microtissue models for research and translational applications.

## Introduction

Human pluripotent stem cells (hPSCs) offer considerable promise as a cell source for regenerative medicine. Traditional 2-dimensional (2D) stem cell culture is suitable for basic research applications but lacks the scalability required for biomanufacturing and the biological complexity required to generate organoids for drug/toxin screening^1,2^. Alternatively, three-dimensional (3D) cell aggregates are an attractive cell culture format for such applications. Stem cell aggregates offer increased surface area for cell growth per media volume, which enables stem cell expansion at the scale required for cell therapies^3^. In addition, cell aggregates applied as implantable “scaffold-free” constructs show enhanced survival and function *in vivo* ^4–7^. Stem cell aggregates also serve as starting materials for generating organoids, complex multicellular constructs that recapitulate structural and functional aspects of human organs and are useful as *in vitro* tissue models for predicting responses to drugs and toxins^8,9^. The process of cell aggregate formation, typically via reaggregation of singularized cells, is a critical initial step for the generation of many organoids. While several types of stem/progenitor cells have demonstrated an intrinsic capacity to “self-organize” into 3D tissue-specific organoids^10,11^, current approaches offer little control over parameters associated with the aggregation process (e.g., aggregate size, shape, formation kinetics), which limits optimization of stem cell expansion/differentiation processes and impedes identification of requisite conditions for organoid formation.

Conventional methods for generating stem cell aggregates, such as hanging drops and spontaneous aggregation (reviewed in^12,13^), are typically low throughput or offer minimal control over properties of resulting aggregates. To address these shortcomings, recent approaches have relied on “forced aggregation”, wherein defined numbers of singularized cells are centrifuged into microwell arrays to form size-controlled aggregates^14,15^. While this strategy has been applied toward scalable production of aggregates of hPSCs and other cell types, functional equivalence to other methods of aggregation has not been well demonstrated, and the centrifugation force applied in these approaches may have unintended effects on stem cell viability and differentiation^16,17^. Despite the importance of stem cell aggregates in bioprocessing applications, few studies have investigated the influence of aggregation parameters on early lineage bias in pluripotent stem cell differentiation. For example, aggregation kinetics may instruct the development of aggregate structural characteristics, thereby altering the microenvironment created within aggregates and the resulting cell phenotype. Since the process of aggregation depends on expression and affinities of cell-cell adhesion molecules such as cadherins, aggregation kinetics are often difficult to systematically modulate without changing the cells’ adhesive properties, e.g., via engineered cell surface modifications^18,19^. Bioengineering strategies have achieved improved control over aggregation kinetics by modulating variables such as rotary speed applied to aggregates maintained in dynamic suspension culture; however, these approaches rely on external manipulations that change hydrodynamic forces^20^ applied to cells, which may have inherent effects on pluripotency maintenance and differentiation. Consequently, there is a need for methods that control cell aggregation kinetics in the absence of external manipulation.

In this study, we developed a bioengineered platform for highly controllable self-assembly of 3D stem cell aggregates from labile synthetic substrates. The tunability of labile substrates enabled control over resulting aggregate parameters, including size, shape, and aggregation kinetics. Using an embryoid body (EB) model, we evaluated the influence of aggregation parameters on hPSC lineage bias, and identified aggregation method and kinetics as parameters that may influence EB structure and indirectly instruct stem cell fate.

## Results

### Labile substrates promoted cell aggregate self-assembly

**Figure 1:**
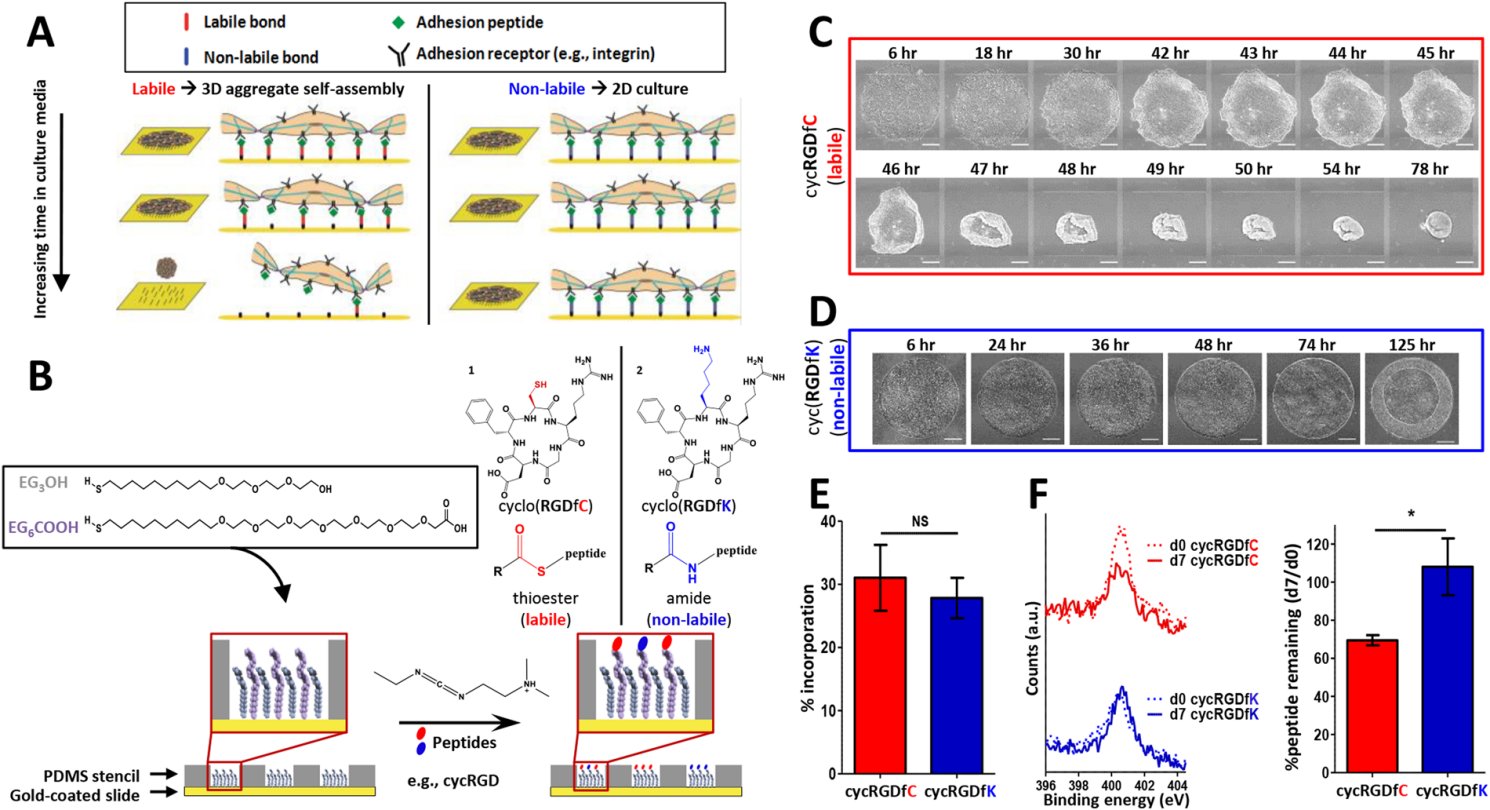
Self-assembly mechanism of stem cell aggregates is dependent on substrate lability. (**A**) Proposed mechanism of cell aggregate self-assembly on substrates presenting labile chemical bonds. Loss of adhesion peptide over time on labile surfaces promotes 3D cell aggregate self-assembly (left) that is not observed on substrates that present non-labile bonds tethering adhesion peptides stably to the surface (right). (**B**) Schematic representation of procedure for forming patterned SAM arrays. (inset) Model peptide adhesion ligands that covalently couple to carboxyl-terminated alkanethiol SAMs to form 1) “labile” thioester or 2) “non-labile” amide linkages between the peptide and the SAM. “R” denotes the EG_6_COOH alkanethiol, excluding the terminal carboxyl group. (**C**) Phase images from timelapse microscopy of hPSC aggregate self-assembly from 2-dimensional monolayers, as shown on 5% cycRGDfC (labile) patterned SAMs. (**D**) Phase images from timelapse microscopy of hPSCs grown on 5% cycRGDfK (non-labile) patterned SAMs, which prohibit aggregate self-assembly. Scale bars in (**C**) and (**D**) represent 250 µm. (**E**) Efficiency of peptide incorporation on cycRGDfC and cycRGDfK SAMs. (**F**) (left) Representative XPS scans of N(1 s) signal on cycRGDfC and cycRGDfK SAMs immediately after SAM functionalization (dashed lines) and after 7-day incubation in media (solid lines). (right) Quantification of percentage of initial peptide remaining on surface after 7-day incubation in media. Values in (**E**) and (**F**) represent the mean ± s.d. of *n* = 3 replicates, *p < *0*.*05*. “NS” denotes no statistical significance.

A bioengineered platform based on alkanethiol self-assembled monolayers (SAMs) enabled self-assembly of 3D cell aggregates from substrates presenting the common cell adhesion peptide RGD via a labile bond (Fig. 1A). Carboxyl-terminated alkanethiols (EG_6_COOH) reacted with nucleophilic functional groups on peptides to covalently link them to the SAM, while substrate peptide density was controlled by changing % EG_6_COOH - the ratio of reactive EG_6_COOH-terminated to bioinert EG_3_OH-terminated alkanethiol groups. Cell adhesion was spatially restricted to patterned islands by reacting EG_6_COOH with cell adhesion peptides in regions designated by a silicone stencil (Fig. 1B). We seeded hPSCs onto patterned 5% EG_6_COOH SAMs reacted with a cysteine-containing cyclic RGD peptide (“5% cycRGDfC”), referred to hereafter as “labile substrates” based on the labile thioester linkage^21–24^ formed via the reaction between cysteine free thiols and EG_6_COOH (molecule 1, Fig. 1B inset). On labile substrates, confluent monolayers of hPSCs detached at the edges of patterned colonies and involuted to form three-dimensional cell aggregates in a process we termed “cell aggregate self-assembly” (Fig. 1C, Supplemental Video 1). To determine whether self-assembly was dependent on bond lability, we also tested a lysine-substituted cyclic RGD variant (cycRGDfK, molecule 2, Fig. 1B inset) to generate non-labile substrates presenting an equivalent surface density of RGD via a stable amide linkage between the SAM and the peptide. As expected, although cycRGDfC and cycRGDfK peptides incorporated into SAMs with similar efficiencies (Fig. 1E), significant peptide loss occurred on labile substrates over a 7-day incubation in cell culture media, while incubation in cell culture media had negligible effect on the peptide content of non-labile substrates (Fig. 1F). Furthermore, hPSCs seeded on non-labile substrates failed to form aggregates and instead remained confined to patterned regions (Fig. 1D).

### Patterning of labile substrates formed size- and shape-controlled EBs

**Figure 2:**
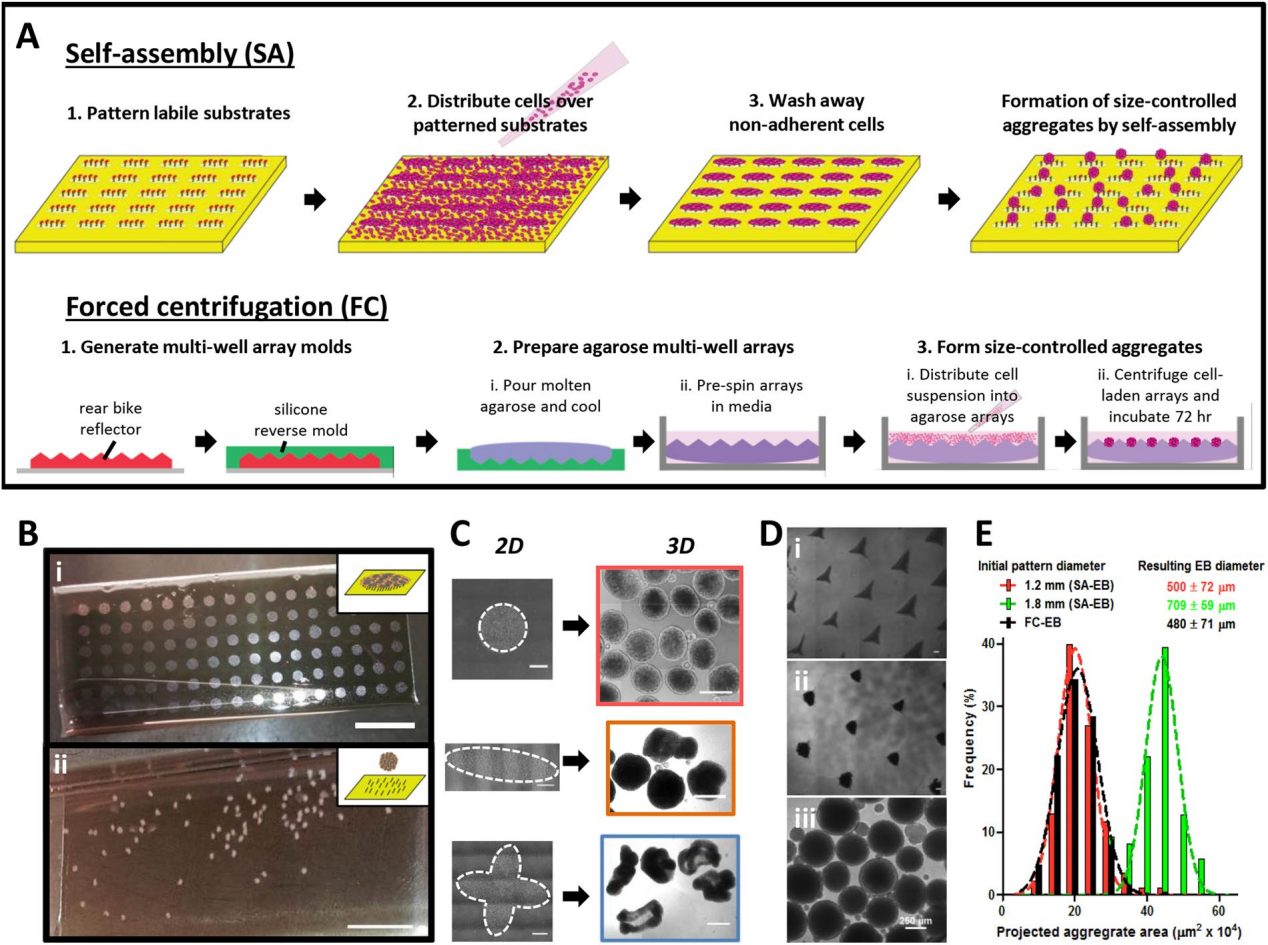
Efficient generation of EBs via self-assembly (SA) and forced centrifugation (FC). (**A**) Schematics demonstrating the process of SA-EB formation on labile substrates (top) and FC-EB formation in agarose microwells (bottom). (**B**) (i) hPSCs seeded on patterned labile substrates remain in 2D at 4 hrs and (ii) self-assemble into 3D aggregates within 72 hrs. (**C**) Patterning of labile substrates enables control over the geometry of hPSC colonies in 2-dimensional culture and leads to the self-assembly of size- and shape-controlled 3-dimensional EBs. (**D**) FC-EBs in agarose microwells at (i) 4 hrs and (ii) 72 hrs after seeding, and (iii) after collection at 72 hrs post-seed. (**E**) Histogram of cross-sectional area for SA-EBs generated from 2-dimensional patterns of varying size. Size distribution of FC-EBs generated by forced centrifugation into microwells is overlaid for comparison (black histogram). Resulting EB diameter is represented as the mean ± s.d. Scale bars represent (**B**) 10 mm, (**C**) 500 μm, (**D**) 250 μm.

Patterning of labile substrates in various geometries (e.g., circles, ovals, quatrefoils of varying dimensions) supported the formation of size- and shape-controlled hPSC colonies in 2-dimensional culture and led to the self-assembly of viable 3-dimensional EBs (Fig. 2A–C, Supplementary Fig. S1). Day 0 self-assembled EBs (SA-EBs) formed from 1.2 mm circles (mean diameter = 500 ± 72 μm) were smaller than those formed from 1.8 mm circles (mean diameter = 709 ± 59 μm), and mean SA-EB diameter correlated with size of the initial 2-dimensional pattern. We compared SA-EBs from circular patterns to EBs formed by forced centrifugation into agarose microwells (FC-EBs), a commonly used method for generating size-controlled aggregates (Fig. 2A)^14,25–27^. hPSCs seeded and centrifuged into microwells started as amorphous clusters and compacted into tight, viable aggregates within 24 hours (Fig. 2D). Similar to what has been observed in other studies employing FC-EBs, not all cells within the microwells incorporated into EBs^26^ and those that did not incorporate were non-viable. Nevertheless, resulting FC-EB size was directly correlated to the initial cell number seeded per microwell (Supplementary Fig. S1). A seeding density of 25,000 cells per microwell generated FC-EBs of similar size distribution (mean diameter = 480 ± 71 μm) to SA-EBs from 1.2 mm circular patterns (Fig. 2E). Therefore, to mitigate potential confounding effects of EB size on differentiation, we chose to use SA-EBs from 1.2 mm patterns and FC-EBs seeded at 25,000 cells per microwell for further comparisons.

### EB formation method instructed pluripotency and lineage bias

**Figure 3:**
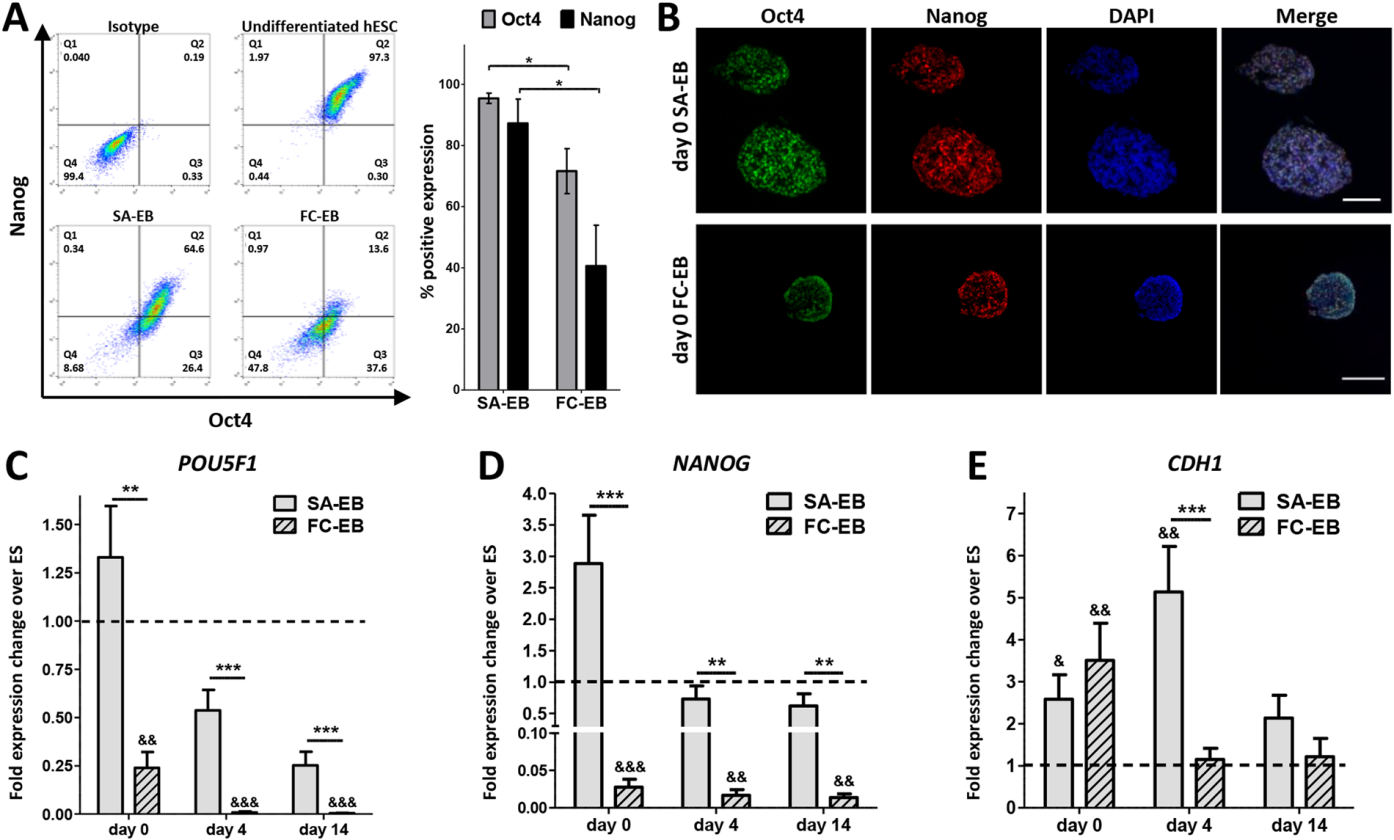
Assessment of pluripotency loss in SA-EBs and FC-EBs during spontaneous embryoid body differentiation. (**A**) Representative flow cytometry plots (left) and quantification (right) showing Oct4 and Nanog expression in day 0 SA-EBs and FC-EBs. Error bars represent s.e.m. from *n* = 4 independent biological replicates. (**B**) Immunofluorescence staining for pluripotency markers Oct4 and Nanog in sectioned day 0 SA-EBs (i) and FC-EBs (ii). Scale bar represents 250 μm. (**C,D,E**) *POU5F1*, *NANOG*, and *CDH1* expression in SA-EBs and FC-EBs at days 0, 4, and 14 during spontaneous EB differentiation. Fold-changes in expression are relative to undifferentiated hPSCs. Values represent the mean ± s.e.m. of *n* = 3 independent biological replicates. Dashed line represents expression level of undifferentiated hPSCs. Asterisks represent statistical significance between indicated conditions (*p < *0*.*05*, **p < *0*.*005*, ***p < *0*.*0005*); &s represent the same levels of statistical significance, relative to undifferentiated hPSCs.

hPSCs retained pluripotency marker expression during cell aggregate self-assembly, with 95.4 ± 3.4% Oct4 + and 87.3 ± 15.8% Nanog + cells in day 0 SA-EBs, as determined by flow cytometry (Fig. 3A, Supplementary Fig. S2). Accordingly, we observed robust expression of Oct4, Nanog, Sox2, and E-cadherin in immunostained aggregate sections, which showed minimal expression of early differentiation markers at day 0 (Fig. 3B, Supplementary Fig. S3). In contrast, both Oct4 and Nanog were rapidly lost in FC-EBs, which were only 71.6 ± 14.7% Oct4 + and 40.6 ± 26.7% Nanog + at day 0. We next subjected SA-EBs and FC-EBs to spontaneous differentiation to determine whether EB formation method biases differentiation trajectory (Supplementary Fig. S4). Consistent with assessment of EBs by flow cytometry, levels of the pluripotency genes *POU5F1* and *NANOG* were markedly reduced in day 0 FC-EBs (0.24-fold and 0.03-fold change relative to undifferentiated hPSCs, respectively) and decreased further in day 4 and day 14 FC-EBs. In contrast, day 0 SA-EBs exhibited comparable expression of *POU5F1* and increased expression of *NANOG* (2.88-fold change) relative to undifferentiated hPSCs (Fig. 3C–D). Expression of both *POU5F1* and *NANOG* remained significantly higher in SA-EBs compared to that of FC-EBs at all equivalent time points. *CDH1* (E-cadherin), a gene associated with pluripotency, was significantly upregulated in both day 0 SA-EBs and FC-EBs compared to undifferentiated hPSCs in 2-dimensional culture. *CDH1* expression in SA-EBs was further upregulated at day 4 before decreasing by day 14, while expression in day 4 and day 14 FC-EBs was not significantly different from that of undifferentiated hPSCs (Fig. 3E, Supplementary Fig. S5).

**Figure 4:**
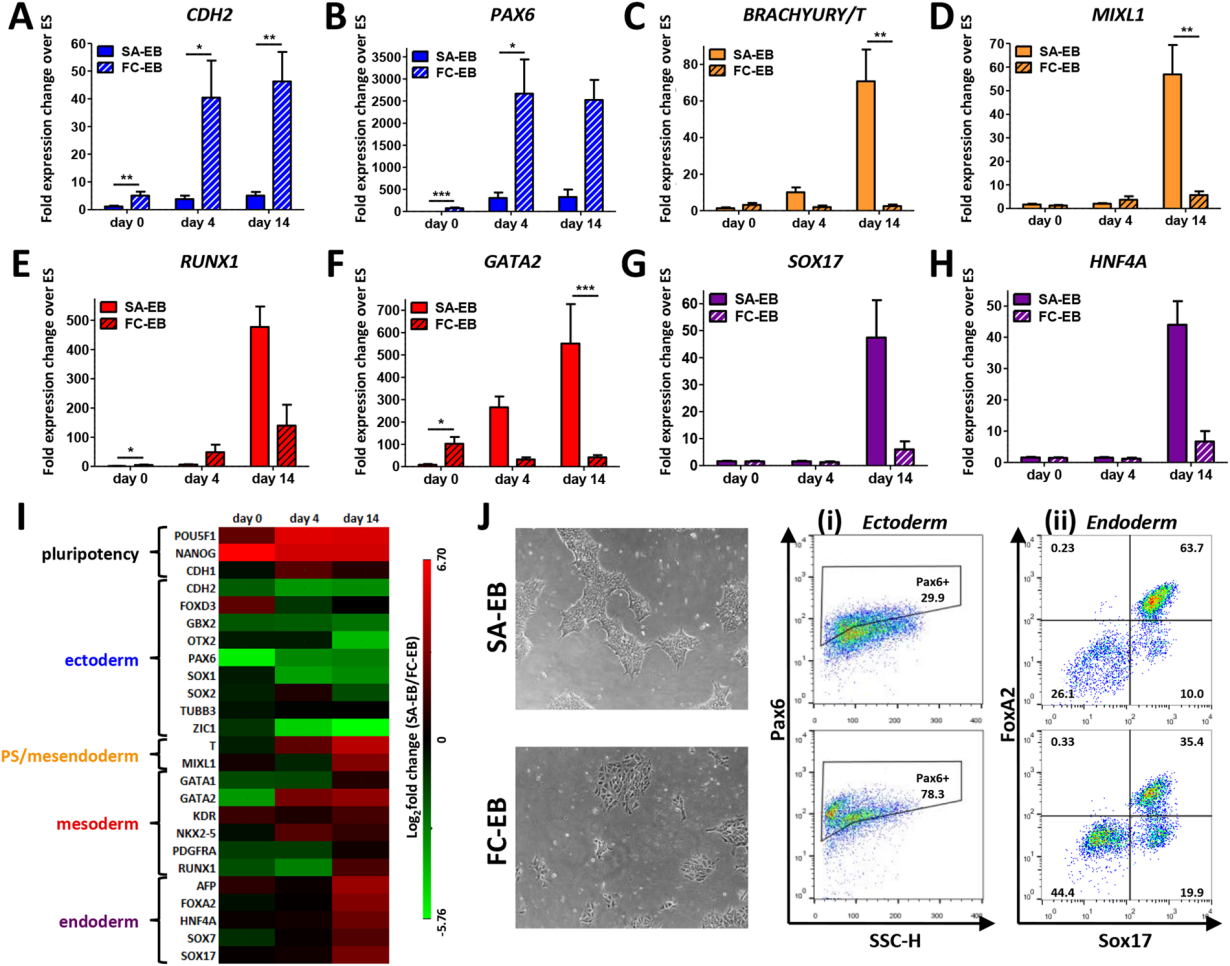
Analysis of the propensity of SA-EBs and FC-EBs to differentiate toward the primary germ lineages. Expression of genes related to ectoderm (**A**,**B**), primitive streak (PS)/mesendoderm (**C**,**D**), mesoderm (**E**,**F**), and endoderm (**G**,**H**) in SA-EBs and FC-EBs at days 0, 4, and 14 during spontaneous differentiation. Fold-changes in expression are relative to undifferentiated hPSCs. Values represent the mean ± s.e.m. of *n* = 3 independent biological replicates. Asterisks represent statistical significance between indicated conditions (*p < *0*.*05*, **p < *0*.*005*, ***p < *0*.*0005*). (**I**) Summary of gene expression related to pluripotency and differentiation toward ectoderm, mesoderm, and endoderm in SA-EBs vs. FC-EBs on days 0, 4, and 14 of spontaneous differentiation, expressed as log_2_(fold expression change of SA over FC). Values represent the mean of *n* = 3 independent biological replicates. (**J**) Directed differentiation of SA-EB- and FC-EB-derived cells. (left) Phase contrast images of cells derived from day 0 SA-EBs and FC-EBs prior to directed differentiation. (right) Flow cytometry plots showing expression of (i) neuroectoderm marker Pax6 and (ii) definitive endoderm markers FoxA2 and Sox17 in cells differentiated from day 0 SA-EBs (top) and FC-EBs (bottom).

The method of aggregate formation significantly influenced lineage bias of the resulting EBs during spontaneous differentiation. Several genes associated with germ layer differentiation were upregulated in FC-EBs relative to SA-EBs at day 0, including genes representative of ectoderm (*CDH2*, *PAX6*), primitive streak (*BRACHYURY/T*), and mesoderm (*RUNX1*, *GATA2*) (Fig. 4A–H). During early differentiation, FC-EBs demonstrated a bias toward ectoderm differentiation, with 8 of 9 ectoderm-associated genes upregulated relative to SA-EBs at day 0, including *PAX6*, which was increased >40-fold in FC-EBs. By day 14, a clear distinction was evident between SA-EBs and FC-EBs; FC-EBs demonstrated a greater preference for ectoderm differentiation while SA-EBs upregulated genes associated with primitive streak, mesoderm, and endoderm differentiation (Fig. 4I). In support of these results, we observed neural rosettes in >50% of plated FC-EBs and the emergence of cells of neuronal morphology at the perimeter of FC-EB outgrowths within 2–3 days following the first appearance of rosettes (Supplementary Fig. S4). We did not observe any neuronal cells in SA-EB outgrowths throughout the course of spontaneous EB differentiation.

Differences in lineage bias in SA-EBs versus FC-EBs were associated with signaling downstream of the transforming growth factor-beta (TGF-β)/Activin/Nodal signaling axis. Histology revealed that phosphorylation of Smad2/3 was homogeneous throughout day 0 SA-EBs, whereas phosphoSmad2/3 was strongly expressed near the periphery but diminished in the interior of FC-EBs. Accordingly, western blot analysis of day 0 EBs showed higher levels of phosphoSmad2/3 in SA-EBs compared to FC-EBs (Supplementary Fig. S6). To determine whether differential levels of Smad2/3 signaling in SA-EBs and FC-EBs correlated with the efficiency of their directed differentiation, we next induced cells dissociated from day 0 EBs toward lineages known to be specified (definitive endoderm)^28,29^ or inhibited (neuroectoderm)^30,31^ by the TGF-β/Activin/Nodal signaling axis. Cells from SA-EBs differentiated with higher efficiency toward definitive endoderm compared to those from FC-EBs (63.7% vs. 35.4% FoxA2/Sox17 +). In contrast, differentiation toward neuroectoderm was less efficient in cells from SA-EBs (29.9% Pax6 +, vs. 78.3% Pax6 + from FC-EBs).

### Fast vs. slow aggregation kinetics correlated with lineage-specific gene expression in spontaneously differentiating EBs

**Figure 5:**
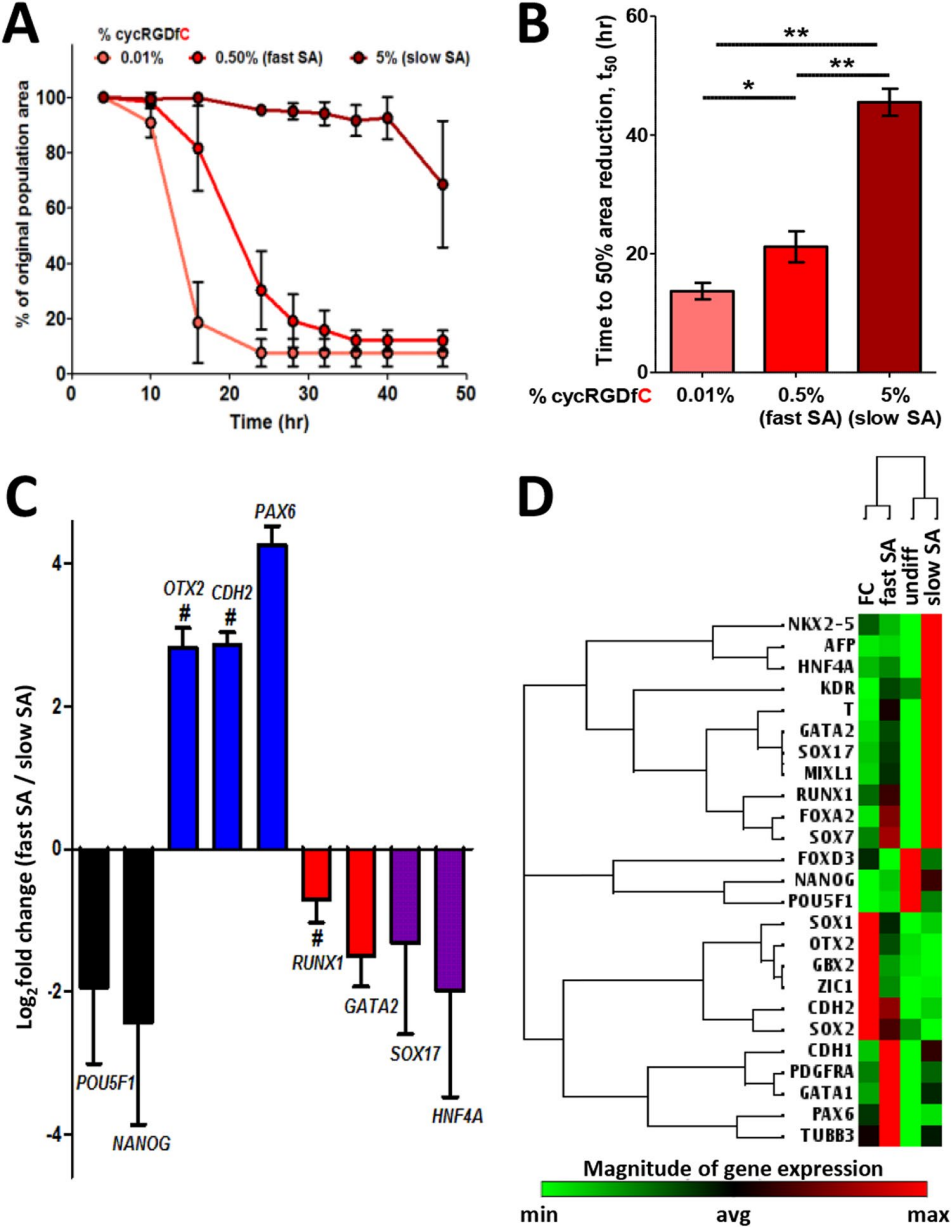
Labile substrate ligand density controls self-assembly kinetics and modulates lineage-specific gene expression during spontaneous EB differentiation. (**A**) hPSC aggregate self-assembly kinetics as a function of initial peptide density on labile substrates. Values represent the mean of *n* = 10, 12, and 6 replicates for 0.01%, 0.5%, and 5% cycRGDfC, respectively. Error bars represent 95% c.i. (**B**) Mean t_50_ values of self-assembly kinetics for EBs generated from labile substrates of varying cycRGDfC density. Error bars represent s.e.m. *p < 0.001, **p < 0.0001. (**C**) Fold-change expression of pluripotency and differentiation genes at day 14 in 0.5% SA-EBs (fast SA) vs. 5% SA-EBs (slow SA). ^#^Represents statistically significant difference between “slow SA” and “fast SA” (*p* < 0.05). Values represent the mean ± s.e.m. of *n* = 3 independent biological replicates. (**D**) Non-supervised hierarchical clustering of day 14 pluripotency and differentiation gene expression for slow and fast SA-EBs, FC-EBs, and undifferentiated hPSCs. Values represent the mean of *n* = 3 independent biological replicates.

We next modulated cycRGDfC peptide density on labile substrates to control the kinetics of cell aggregate self-assembly from labile substrates. Specifically, we tracked the projected area of individual patterned cell populations over time and determined a t_50_ value for each (defined as the time required for the cell monolayer to assemble into 3D and decrease its surface coverage to 50% of the original 2D monolayer population area) as a metric by which to quantify aggregation kinetics (Supplementary Fig. S7). The rate of aggregate self-assembly was dependent on initial peptide density, with hPSCs on 0.01% cycRGDfC SAMs reaching t_50_ by 14 hours while hPSCs on 0.5% and 5% cycRGDfC exhibited mean t_50_ values of 21 hours and 46 hours, respectively (Fig. 5A–B). In line with our previous observations, hPSCs on SAMs presenting a similar range of cycRGDfK densities (non-labile substrates) did not undergo self-assembly to form aggregates.

Fast vs. slow aggregation kinetics resulted in distinct profiles of lineage-specific gene expression in differentiating EBs. We assessed EBs formed from 0.5% cycRGDfC substrates (“fast SA-EB”), which self-assemble with accelerated kinetics compared to those formed from 5% cycRGDfC substrates (“slow SA-EB”). We considered FC-EBs as representing the extreme end of the spectrum associated with fastest aggregation kinetics, as our observations corroborated previous reports of FC-EB formation within 12–24 hours^26^. By day 14, fast SA-EBs exhibited increases in ectoderm gene expression (e.g., *OTX2*, *CDH2*, and *PAX6*) and decreases in expression of pluripotency (*POU5F1*, *NANOG*), mesoderm and endoderm genes (e.g., *RUNX1*, *GATA2*, *SOX17*, and *HNF4A*), relative to slow SA-EBs (Fig. 5C). For 18 of the 25 pluripotency and differentiation genes assessed, fast SA-EBs represented an intermediate expression profile between slow SA-EBs and FC-EBs (Fig. 5D), and non-supervised hierarchical clustering resulted in the grouping of fast SA-EBs with FC-EBs. These data suggested that the observed bias in EB differentiation trajectory may be a function of aggregation kinetics.

**Figure 6:**
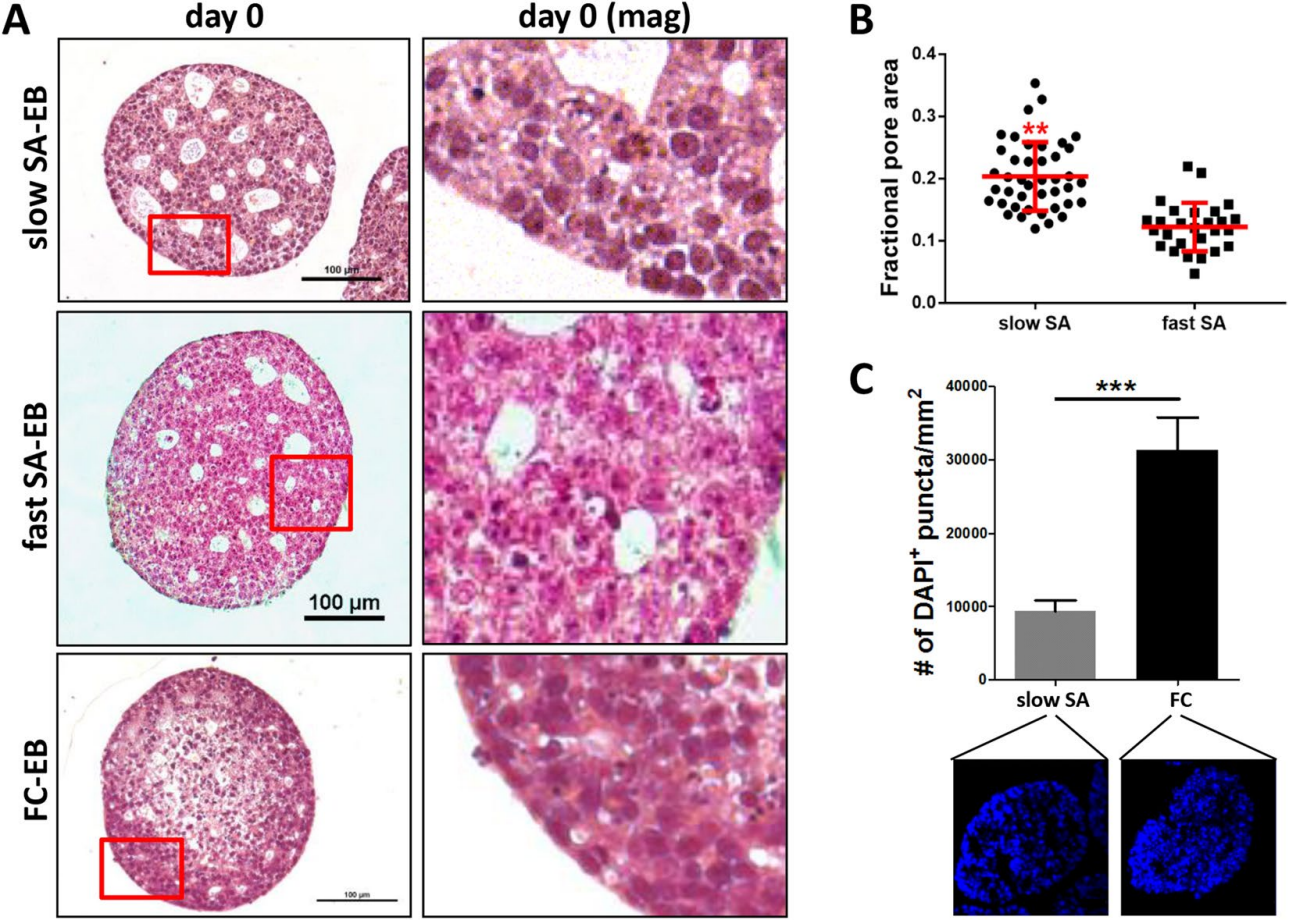
Aggregation method and kinetics affect EB structure. (**A**) H&E staining of histological sections from day 0 slow SA-EBs, fast SA-EBs, and FC-EBs. (right) Higher magnification micrographs of representative areas from EB sections (red boxes, left). (**B**) Quantification of porosity in slow and fast SA-EBs. Individual points represent values analyzed from histological sections of *n* = 6 distinct EBs per condition. Red bars represent the mean ± s.d. (**p < 0.01, two-tailed Student’s t-test) (**C**) Quantification of cell density in cryosectioned day 0 SA-EBs and FC-EBs, as represented by number of DAPI-positive puncta per area. Values represent the mean ± s.d. (***p < 0.0005, two-tailed Student’s t-test).

The timing and kinetics of morphogenesis events have been associated with changes to tissue structure and function in developmental contexts (e.g., neural tube closure, palate development, mesenchymal condensation) as well as in 3D spheroid models *in vitro* ^32–34^. To determine whether differences in aggregation kinetics affected subsequent aggregate structure, we assessed slow SA-EBs, fast SA-EBs, and FC-EBs by histology. FC-EBs exhibited a compacted morphology with densely packed cells and minimal internal porosity. In contrast, SA-EBs displayed extensive porosity, with large pores distributed throughout the interior of slow SA and fast SA-EBs (Fig. 6A). Image analysis of H&E-stained histological sections showed that slow SA-EBs were more porous than fast SA-EBs (Fig. 6B), as measured by the fraction of EB area occupied by pores (0.20 ± 0.03 vs. 0.12 ± 0.03 for slow vs. fast SA-EBs, respectively; *p* < 0.01). Day 0 SA-EBs were also less cell-dense compared to FC-EBs, with fewer DAPI + puncta per EB area (Fig. 6C). These initial results suggested that the observed bias in EB differentiation trajectory may be a function of changes to EB structure, aspects of which may also be influenced by aggregation kinetics.

## Discussion

In this study, we patterned chemically defined labile substrates to generate size/shape-controlled EBs that self-assemble in the absence of physical manipulation or enzymatic treatment. We demonstrated that substrates formed via the reaction of free thiol-containing RGD cell adhesion peptides with carboxylate-presenting surfaces (“labile substrates”) would promote the collective assembly of 2-dimensional cell cultures into 3-dimensional cell aggregates (“cell aggregate self-assembly”) through a controllable, lability-dependent process (Fig. 1A).

Several seminal studies have reported that EB size^35–38^ is a major determinant of lineage bias during spontaneous differentiation. In light of these considerations, we attempted to limit EB size-dependent effects on differentiation by optimizing seeding conditions for each aggregation method, resulting in size-matched EBs between the SA and FC methods (Fig. 2). While the resulting FC-EBs resembled SA-EBs in size and shape, FC-EBs exhibited a rapid and drastic loss of pluripotency marker expression and demonstrated a bias toward ectoderm lineages that was apparent as early as day 0 and persisted in later stages of spontaneous differentiation (Figs 3 and 4). In contrast, EBs formed via self-assembly from labile substrates exhibited an initial delay in the loss of pluripotency marker expression and had a higher propensity to generate mesoderm and endoderm derivatives. These results indicate that formation method in size-matched EBs has a substantial effect on early lineage bias. Importantly, our findings may partly explain the disparities between numerous reports that have investigated lineage bias solely as a function of EB size. Indeed, it is common practice to stratify initial EB populations based on morphometric traits such as EB diameter^14,15,35,36,39^; however, such metrics give little indication of the processing parameters that affect cell fate prior to and during EB formation. Based on the results of this study, aggregation method and kinetics should be considered as critical parameters when developing bioprocessing workflows for the expansion of hPSC aggregates and in the development of stem cell-derived organoids.

Strategies to prevent differentiation of stem cell aggregates while retaining the scale-up and bioprocessing advantages of 3D culture are of particular interest for cell therapy applications^1,40^. Compared to 2D monolayer culture, suspension culture of hPSCs as aggregates enables more efficient use of nutrients and growth factors in the media during scale-up cell expansion. However, restricted transport of oxygen and media components into the interior of hPSC aggregates can lead to stem cell population heterogeneity and the onset of spontaneous differentiation, which reduces downstream differentiation efficiency and yield of desired cell types. Pluripotency markers such as Oct4 and Nanog are often downregulated rapidly in EBs of comparable size (~400–500 μm diameter) to those used in this study, an effect that has been partially attributed to limited diffusion of pluripotency-promoting cytokines into the interior of large, cell-dense aggregates^41–43^. Notably, we found that aggregation method alone influences pluripotency marker loss in EBs, with SA-EBs exhibiting sustained expression of Oct4 and Nanog compared to stage-matched FC-EBs. One possible explanation for this result is that the aggregate self-assembly process results in a more porous EB structure with improved mass transport characteristics, which may promote access of nutrients and signaling molecules from culture media into the EB interior. In particular, early SA-EBs exhibited a highly porous structure, low cell density, and minimal necrosis despite EB diameters exceeding 200 μm, in stark contrast to the dense cell packing, interior cell death, and necrotic core formation we observed in FC-EBs (Fig. 6, Supplementary Fig. S8).

Importantly, we also found that in early EBs cultured in Essential 8 medium, in which TGF-β is a critical component for pluripotency maintenance^44^, TGF-β-dependent Smad2/3 phosphorylation was homogeneous throughout the aggregate interior in SA-EBs but decreased as a function of distance from the aggregate perimeter in FC-EBs. This expression pattern was not only consistent with the spatial distribution of Oct4 and Nanog but also corresponded to higher overall TGF-β signaling activity in SA-EBs compared to FC-EBs (Supplementary Fig. S6). The TGF-β signaling pathway has a well-established role in hPSCs, where activation of downstream Smad2/3 transcriptional regulators has been shown to be essential for pluripotency maintenance and mesendoderm specification whereas inhibition of TGF-β signaling is required for efficient neural differentiation^31^. Our observation that highly porous SA-EBs showed high TGF-β activity and mesoderm/endoderm differentiation, while decreased porosity in FC-EBs correlated with low TGF-β activity and ectoderm differentiation, further supports the possibility that differences in soluble factor transport are responsible for the distinct lineage biases observed between EBs formed via different aggregation methods and kinetics. In light of these findings, we speculate that the process of self-assembly from labile substrates promotes the emergence of unique structural and functional properties not previously exhibited in stem cell aggregates, which may in turn affect the transport and signaling of soluble factors that instruct cell fate within the aggregate. Future studies will aim to better understand the implications of these properties for downstream applications of cell aggregates in stem cell biomanufacturing and the generation of organoids.

In addition to the effects of aggregation method on the timing of pluripotency marker loss in EBs, we found that aggregation method also has a significant impact on EB lineage bias during spontaneous differentiation. The propensity for SA-EBs to preferentially generate mesendoderm derivatives during later stages of differentiation may be related to their high levels of *POU5F1* (Oct3/4) and *NANOG* expression at the start of differentiation, as both were upregulated in day 0 SA-EBs compared to undifferentiated hPSCs (Fig. 3). Although Oct4 and Nanog are known to cooperate in a self-regulating pluripotency network with Sox2, they fulfill distinct roles as both pluripotency-promoting factors and lineage specifiers^45–47^. Multiple studies suggest that high Nanog levels reinforce the pluripotent state; Nanog overexpression permits pluripotency maintenance in the absence of leukemia inhibitory factor in mouse embryonic stem cell culture^48^ and enables the robust growth of undifferentiated hPSCs in feeder-free cultures^49^. On the other hand, elevated levels of Oct4 expression have been linked to lineage priming toward mesendoderm fates^47,50,51^. Ramanathan and coworkers previously reported that Oct4 and Sox2 levels can serve as continuous temporal markers of hPSC progression toward lineage selection prior to the activation of lineage-specific markers; Oct4 was found to specifically repress only the neural ectoderm fate, while Sox2 repressed only the mesendoderm fate^52^. In the present study, we observed ~2-fold lower *SOX2* and ~5.5-fold higher *POU5F1* expression in day 0 SA-EBs compared to FC-EBs (Fig. 4), which in combination may push cells toward mesendoderm bias. Of note, the population-level changes in gene expression reported here likely do not capture the full extent of gene expression differences and lineage bias exhibited by cells as a function of their spatial location within SA-EBs or FC-EBs. Single cell analyses, particularly in combination with emerging technologies that allow for spatial resolution of gene expression^53,54^, will be crucial for understanding how cell aggregation parameters influence spatiotemporal patterning within stem cell-derived microtissues and its consequences for organoid culture.

An increasing interest in dictating the structural and functional properties of developing 3D human organoids has led to technologies that allow improved control over various aspects of 3D cell aggregation. Aggregation parameters that may be controlled include the composition of the initial (stem) cell population, the mechanism of aggregation, the size and shape of resulting aggregates, and the presence of exogenous or cell-secreted factors. Aggregation kinetics constitute an additional parameter that has previously been suggested to influence stem cell differentiation trajectory^20,33,55^, but could not be evaluated without introducing variables such as hydrodynamic forces^20^ that may have inherent effects on differentiation^1^. In this study, we demonstrated that labile substrates could be “pre-programmed” to control aggregation kinetics without the requirement for physical or enzymatic perturbations, and found that changing aggregation kinetics resulted in differences in EB lineage bias, with faster aggregation promoting ectoderm and inhibiting mesendoderm fates during EB differentiation.

One mechanism by which altered aggregation kinetics may influence stem cell fate is through signaling downstream of E-cadherin adherens junctions. It is well established that E-cadherin junctional complex formation is dependent on the extent of cell-cell contacts, which increases when cells are grown as 3D aggregates versus 2-dimensional culture^20,56^. In our study, while *CDH1* was upregulated in all day 0 EBs relative to monolayer hPSCs, the temporal profile of *CDH1* expression varied depending on EB aggregation kinetics (Supplementary Fig. S5). In FC-EBs, which formed compact aggregates within 12 hours after seeding, *CDH1* levels peaked at day 0 and decreased thereafter, whereas slow SA-EBs exhibited an increase in *CDH1* between days 0 and 4 of differentiation before gradual loss of expression. Similarly, Kinney *et al*. reported that when rotary speed was modulated to change aggregation kinetics of mouse EBs, rapidly aggregating EBs had monotonically decreasing E-cadherin expression over 7 days of differentiation while slower aggregating EBs exhibited biphasic expression levels that peaked at mid-differentiation^55^. The kinetics of E-cadherin expression was further linked to Wnt/β-catenin signaling activity, which was subsequently found to affect the efficiency of cardiogenic differentiation. Interestingly, we found that expression kinetics of the Wnt target gene *FZD7* matched the trajectory of *CDH1* expression in both SA-EBs and FC-EBs (Supplementary Fig. S5). Further studies are needed to establish a mechanistic link between the temporal regulation of E-cadherin expression and differentiation outcomes downstream of Wnt and other signaling pathways.

It is important to note that, even though we controlled EB size and aggregation kinetics while limiting potential effects on differentiation due to external forces during EB formation, there remain several potentially confounding variables in the comparison of EBs generated via different aggregation methods and kinetics. One inherent difference between EBs formed by different methods is the subsequent culture conditions under which the cells are maintained. In this study, we observed stark differences in the structure of EBs from different formation methods, which results in disparities in effective cell density (Fig. 6) and likely in other properties (e.g., levels of paracrine factors or oxygen gradients within the EBs) that are known to influence stem cell phenotype^43,57,58^. We suspect that many such properties of the microenvironment *within* the EB interior are intrinsic to the method of EB formation and would be difficult to control for without modifying the EB formation method or affecting other parameters (e.g., EB size). As such, the development of aggregation approaches that allow variables such as cell packing density, aggregate size, and oxygen levels to be systematically covaried will be valuable to our understanding of the factors that influence cell fate and phenotype within stem cell aggregates.

The culture environment *external* to EBs is another variable affecting stem cell fate, that may be changed (whether it be intentionally or unintentionally) between different aggregation methods. It is often difficult to control for all differences in the external environment, particularly when the mechanism for EB formation places restrictions on the culture conditions. One obvious example of this is with EBs formed in conventional hanging drops, where the media volume per EB is limited by surface tension, often resulting in depletion of nutrients from the media and changes in EB gene expression within days^16^. In probing the effects of initial culture conditions on EBs, we found that incremental changes in the media volume per cell directly correlated with changes in day 0 *NANOG* expression, independently of the aggregation method used (Supplementary Fig. S9). However, *NANOG* expression in day 0 FC-EBs did not reach levels equivalent to that in SA-EBs when media volume per cell was matched between conditions or even when FC-EBs were provided with >2.5-fold excess media per cell, suggesting that differences in external media conditions alone do not account for the dramatic changes in gene expression that we observed between aggregation methods (Supplementary Fig. S9). In addition, we compared FC-EBs to “settled EBs” that were formed in the same agarose microwell format by gravity-based settling (i.e., without centrifugation) and therefore experienced the same culture conditions as FC-EBs. We found that in comparison to FC-EBs, settled EBs display a similar yet distinct expression profile of lineage-specific genes. At day 14 of differentiation, both FC-EBs and settled EBs showed low levels of the pluripotency genes *POU5F1* and *NANOG* and upregulated expression of several ectoderm-associated genes (e.g., *CDH2*, *SOX1*, *ZIC1*). However, settled EBs also expressed several mesoderm- (*PDGFRA*, *RUNX1*) and endoderm-specific genes (*HNF4A*, *AFP*) at similar levels compared to SA-EBs (Supplementary Fig. S9), providing further evidence that changes to the aggregation method are sufficient to shift EB differentiation under equivalent culture conditions.

Finally, by developing labile substrates that promote cell aggregation via mechanisms fundamentally distinct from existing approaches, we identified porosity within EBs as a variable that likely influences gene expression during their differentiation. EBs formed by conventional methods (gravity-based settling or forced aggregation into microwells) demonstrated minimal porosity while cell aggregate self-assembly from labile substrates supported the extensive development of pores, typically tens of microns in diameter, throughout the interior of EBs (Fig. 6). The origin of pore formation in SA-EBs is currently unknown but appears distinct from what has been previously described for cystic EBs^59,60^, as we have observed the early stages of pore development even prior to the completion of EB self-assembly (not shown) whereas cyst formation is typically observed in later stages of EB differentiation. Interestingly, changing aggregation kinetics by varying the amount of RGD ligand presented on labile substrates had a significant effect on SA-EB porosity, with faster assembling SA-EBs exhibiting decreased porosity compared to slower assembling SA-EBs. Aggregation kinetics also influenced the expression of lineage-specific genes in differentiating SA-EBs, where faster self-assembly favored an ectoderm differentiation program at the expense of mesendoderm fates. Whether these changes in EB differentiation fate are due primarily to differences in aggregation kinetics or porosity, or a combination of both factors, remains to be seen. Nevertheless, our findings suggest that aggregation method and kinetics are critical determinants of EB lineage bias, and open new avenues for studying the relationship between aggregation conditions, structure, and function in stem cell aggregates.

## Conclusions

In the current study, we developed patterns of RGD-presenting labile substrates for self-assembly of human embryoid bodies. Cell aggregate self-assembly from 2D to 3D was dependent on substrate lability, and patterning of labile substrates enabled control over the size and shape of EBs formed. Comparison of EBs generated by self-assembly to those generated by forced centrifugation revealed that the method of aggregation had potent effects on pluripotency maintenance and lineage bias toward neuroectoderm versus mesendoderm during spontaneous and directed differentiation. While there exist numerous methods for generating size-controlled EBs, cell aggregation driven by substrate lability constitutes a novel mechanism for EB formation, in which parameters such as aggregation kinetics can be finely controlled. We modulated aggregation kinetics of self-assembled EBs by varying initial RGD density on labile substrates and found that the rate of aggregation influences lineage-specific gene expression during differentiation. Of note, the self-assembly mechanism allows for control over aggregation kinetics independently of confounding external factors such as physical force, enabling a clearer mechanistic understanding of the effects of aggregation kinetics on cell fate. While the present study provides a proof of concept in EBs generated from uniform hPSC populations, we speculate that the method and rate of aggregation will affect phenotypic outcomes in other cell types, likely in part by altering aggregate structure. Thus, we anticipate that the technological advances and insights described in this study will inform strategies for the biomanufacturing of homotypic and heterotypic cell aggregates for multiple applications, including clinical-scale stem cell expansion and organoid generation.

## Materials and Methods

### Materials and reagents

Carboxylic acid-terminated hexa(ethylene glycol) undecanethiol (HS-C_11_-(O-CH_2_-CH_2_)_6_-O-CH_2_-COOH) (referred to herein as “EG_6_COOH”) and 11-tri(ethylene glycol)-undecane-1-thiol (HS-C_11_-(O-CH_2_-CH_2_)_3_-OH) (referred to herein as “EG_3_OH”) were purchased from Prochimia. N-hydroxysuccinimide (NHS), *n*-(3-dimethylaminopropyl)-N′-ethylcarbodiimide hydrochloride (EDC) and sodium dodecyl sulfate (SDS) were purchased from Fisher Scientific. Cyclo(Arg-Gly-Asp-D-Phe-Cys) (cycRGDfC) and cyclo(Arg-Gly-Asp-D-Phe-Lys) (cycRGDfK) peptides were purchased from Peptides International and used in SAM experiments at a concentration of 0.3 mM in pH 7.4 PBS unless otherwise indicated.

### Fabrication of PDMS stencils

Polydimethylsiloxane (PDMS) stencils containing arrays of wells were fabricated by soft lithography^61^. Briefly, master molds containing arrays of cylindrical posts were fabricated from SU-8 spin-coated silicon wafers using conventional photolithography techniques. PDMS was prepared by mixing a 10:1 ratio of base to curing agent, followed by degassing for >45 minutes. The degassed mixture was cast over the master and cured for 6 hr at 80 °C. Following curing, PDMS stencils were removed from molds and cleaned in hexanes using an overnight Soxhlet extraction. Stencils were allowed to dry in a fume hood for at least 2 hours prior to use.

### Preparation of SAMs

We used a mixed self-assembled monolayer (SAM) system previously established in our laboratory for generating patterned arrays of alkanethiols on gold with defined and controllable peptide density in each array spot^62^. Hydroxyl-terminated alkanethiols (“EG_3_OH”) served as a bioinert background preventing non-specific protein adsorption, while carboxyl-terminated alkanethiols (“EG_6_COOH”) allowed for conjugation of cyclized RGD (“cycRGD”) cell adhesion peptides via carbodiimide chemistry. Individual conditions within the array were isolated by patterning alkanethiols within an elastomeric stencil, and total peptide density in each array spot was varied by changing the percentage of reactive EG_6_COOH available for peptide coupling among background non-reactive EG_3_OH molecules. Alkanethiol solutions were prepared by combining 1 mM ethanolic solutions of EG_3_OH and EG_6_COOH at molar ratios equivalent to the desired surface concentration of EG_6_COOH (e.g., alkanethiol solutions for 5% EG_6_COOH SAMs were composed of 5 EG_6_COOH:95 EG_3_OH volume ratios). For cell-based experiments, SAMs were patterned into 1.2 mm diameter circular spots unless otherwise indicated. 100% EG_6_COOH SAMs were used for all XPS surface analysis experiments.

Gold-coated glass slides (100 Å Au 〈111〉, 20 Å Ti adhesion layer; Platypus Technologies) were cleaned via sonication in 100% EtOH for 2 minutes, rinsed with EtOH, and dried with N_2_ gas. SAM arrays were patterned using PDMS stencils as follows: Briefly, PDMS wells were filled with 1 mM ethanolic alkanethiol solution and incubated for 10 minutes for local SAM formation. Alkanethiol solutions were then aspirated and wells were rinsed with deionized water (diH_2_O). Carboxylate groups were converted to active ester groups by incubating PDMS wells in a solution of 100 mM NHS and 250 mM EDC in diH_2_O for 15 minutes. After an additional rinse with DIUF H_2_O, peptides were covalently coupled to patterned SAMs by incubating peptide solutions in PDMS wells for 1 hour. After peptide conjugation, PDMS wells were rinsed with diH_2_O, and regions surrounding array spots were backfilled by removing the PDMS stencil and incubating the gold substrate with EG_3_OH (0.1 mM in diH_2_O, pH 2) for 10 min. The array was then rinsed with 0.1% SDS, diH_2_O, and EtOH, and dried with N_2_ gas. SAM arrays were stored in 100% EtOH and used within 24 hours of fabrication. Prior to cell-based experiments or cell-free incubation experiments, SAM arrays were incubated in 70% EtOH for 20 min and rinsed with sterile deionized water before placing into cell culture media.

### hPSC maintenance

H1 human embryonic stem cells (hESCs, WA01-DL-12, WiCell) were maintained on Matrigel-coated 6-well plates (8.7 μg/cm^2^) in Essential 8 medium with daily media exchange, and passaged by standard protocols^63^ using Versene-EDTA every 3 to 4 days. Initial hPSC populations for both EB formation methods were >95% Oct4 + and Nanog + by flow cytometry. Initial karyotypic analysis and mycoplasma testing of the WA01 line were provided by WiCell and Bionique, respectively, and demonstrated normal karyotype and no contamination. Authentication of the WA01 line by STR was performed by the UW Molecular Diagnostics Laboratory and demonstrated positive identity.

### Generation of self-assembled embryoid bodies (SA-EBs)

For single-cell seeding of hPSCs onto SAM arrays, cells were washed with PBS and incubated with TrypLE at 37 °C for 5 minutes to singularize cells. Following singularization, cell suspensions were diluted with 2X volume of E8 supplemented with 5 μM ROCK inhibitor (Y-27632) and pelleted by centrifugation at 200 g for 5 minutes. Cell pellets were resuspended in E8 supplemented with 5 μM Y-27632 before seeding at desired densities. After 2 hrs incubation in a humidified incubator at 37 °C and 5% CO_2_ to allow cell adhesion, seeded SAM arrays were immersed in basal medium to remove loosely or non-specifically adhered cells. SAM arrays were then placed into new wells containing fresh E8 medium supplemented with 5 μM Y-27632 and maintained in this medium unless otherwise indicated. High seeding densities were used to ensure confluence shortly after seeding and to minimize defects in the initial cell monolayer. Unless otherwise stated, hPSCs were seeded onto SAMs at a density of 225,000 cells/cm^2^. As the formation of compact and morphologically defined SA-EBs from 5% cycRGDfC substrates occurred within 72 hours after seeding, we denoted this time point “day 0” (Supplementary Fig. S4).

### Generation of forced centrifugation embryoid bodies (FC-EBs) and settled EBs

Agarose microwells were fabricated based on previously published methods^25^. A 85 mm bicycle retro-reflector (Grote 4005/4006) was fashioned into 35 mm diameter circular portions, each of which was used as a template upon which hydrophilic silicone (1:1 mixture of Hydrosil A and B, Siladent) was cast and cured at 40 psi for 180 min to create a reverse mold sized to fit a standard 24-well plate format. Molds were autoclaved prior to each usage. 1.5% agarose in deionized water was sterilized by autoclaving and heated until molten prior to dispensing into silicone reverse molds. Individual agarose molds containing microwells were allowed to solidify at room temperature, transferred to 24-well plates using a sterile spatula, submerged under 1.0 mL/well of E8 supplemented with 10 μM Y-27632, and spun down at 2,000 g for 3 min to remove air trapped underneath the molds.

For single-cell seeding of hPSCs into microwell arrays to form FC-EBs, cells were pre-treated for 4–6 hours with E8 supplemented with 10 μM Y-27632 based on previous results demonstrating extensive cell death and poor formation of FC-EBs without pre-treatment. Cells were then washed with PBS and incubated with TrypLE at 37 °C for 5 minutes to singularize cells. Cell suspensions were diluted with 2X volume of E8 supplemented with 10 μM Y-27632 and pelleted by centrifugation at 200 g for 5 minutes. A small aliquot of cell suspension was used to determine cell count. Cells used to form FC-EBs were resuspended and seeded into agarose molds for a final seeding density of 25,000 cells per EB unless stated otherwise (note that not all cells incorporated into FC-EBs; Supplementary Fig. S1). Plates were centrifuged at 300 g for 5 min (FC-EB only) and incubated at 37 °C, 5% CO_2_. Settled EBs were formed in the same manner, without centrifugation. A half-volume medium exchange with E8 was performed at 24 hrs post-seed (“day −2”, Supplementary Fig. S4).

For media volume:cell number matching control experiments, FC-EBs were formed by seeding hESCs for one EB per well into 96-well polyHEMA-coated roundbottom plates, where media volume per cell could be varied over the range described in Supplementary Fig. S9. Plates were centrifuged at 300 g for 5 min and incubated at 37 °C, 5% CO_2_, and EBs were collected at day 0 for assessment of *NANOG* expression by qPCR.

### Spontaneous differentiation of EBs

EBs were subjected to spontaneous differentiation following a protocol adapted from existing methods^64–67^. EBs were collected at day 0, transferred to suspension culture dishes, and transitioned from E8 to differentiation medium (DM) from day 0 to day 3 (Supplemental Fig. 4); EBs were placed in 75:25 E8:DM at day 0, 50:50 E8:DM at day 1, 25:75 E8:DM at day 2, and 100% DM at day 3, and maintained in DM thereafter. DM consisted of 20% Knockout Serum Replacement in Knockout-DMEM with 0.1 mM β-mercaptoethanol, 1% non-essential amino acids, and 1% L-glutamine. At day 4, EBs were transferred to Matrigel-coated plates and allowed to form adherent outgrowths. Media was changed on adherent EBs every 2 days.

### XPS analysis of SAMs

X-ray photoelectron spectroscopy of peptide-conjugated SAMs was performed using a Thermo Scientific Model K-Alpha XPS instrument equipped with a monochromatic Al Kα X-ray source (*hv* = 1486.7 eV). Survey and high-resolution spectra were obtained using analyzer pass energy of 200 eV and 50 eV, respectively, with an X-ray spot size of 400 μm. High-resolution spectra were obtained for carbon, nitrogen, sulfur, oxygen, and gold. At least three independent sample replicates were scanned per condition. Spectra were analyzed using Thermo Scientific Avantage XPS software package and peak fitting using a Shirley/Smart type baseline. For each sample, measured atomic percent values for the N1(s) and C1(s) peaks were used to determine the N/C ratio, which was used to calculate percentage peptide incorporation at day 0 for cycRGDfC and cycRGDfK surfaces, based on the theoretical N/C ratio for each surface assuming 100% reaction efficiency with the SAM. Mean values of N/C ratio measured from day 7 surfaces were normalized to day 0 values for each respective peptide, and presented as percentage peptide remaining on the surface. Statistical significance was determined by two-tailed Student’s *t*-test (*p* < 0.05).

### Cell-free SAM incubations

All cell-free incubations were performed at 37 °C, 5% CO_2_ to mimic cell culture conditions. SAMs were prepared and sterilized as described above, and incubated in E8 media for 7 days prior to analysis by XPS. Extra SAMs were prepared and immediately used for baseline (day 0) XPS measurements. For each peptide, percentage of peptide remaining (d7/d0) was calculated by dividing the mean N1s peak area from day 7 (post-incubation) samples by the mean N1s peak area from day 0 (pre-incubation) samples.

### Quantification of hPSC aggregation kinetics, EB size distribution, and EB porosity

*hPSC aggregation kinetics during EB self-assembly*. All timelapse images were acquired using a Nikon Ti Eclipse inverted microscope (10X PhL objective) equipped with NIS Elements software, Perfect Focus System, and a TIZ Tokai Hit incubated stage that was humidified and maintained at 37 °C and 5% CO_2_. All image analysis was performed with NIS Elements analysis software. To aid in exploration of cellular and material parameters influencing cell aggregate self-assembly, timelapse images of self-assembling aggregates were subjected to edge detection analysis to track the projected population area over time. The “Autodetect ROIs” feature was used to draw ROIs representing projected population area for each timelapse frame. At each time point, projected population area for each individual spot was normalized to the initial seeded area of the spot to standardize this metric for different starting pattern sizes. For each individual spot, normalized projected population area over time was plotted in MATLAB, fitted to a sigmoid curve using MATLAB’s Curve Fitting Toolbox, and t_50_ values were calculated as the time to reach 50% of initial population area (defined as population area at 4 hrs after seeding). Individual spots for which the projected population area at 4 hrs after seeding was <50% of the theoretical pattern area occurred in only a small fraction of all samples; these samples were omitted from analysis based on our pre-established findings that <50% cell coverage of patterns impeded controllable aggregate self-assembly. Statistical significance between conditions was determined by two-tailed Student’s *t*-test (*p* < 0.05).

#### EB size distribution

SA-EBs and FC-EBs were collected at day 0 and counted from brightfield images. NIS Elements analysis software was used to determine cross-sectional projection areas of individual EBs. EB diameter was calculated from cross-sectional projection areas by assuming spherical EB morphology.

#### EB porosity

EBs were processed for histology (see below), stained with hematoxylin and eosin, and slides imaged on a Nikon Ti Eclipse microscope equipped with DS-U3 color camera and analyzed in Nikon NIS Elements software. Briefly, an ROI was defined for the area of each EB and binary thresholding was applied to highlight the stained area within each EB (“interior ROI”). The total pore area (total ROI area minus thresholded interior ROI area) was normalized to EB area and expressed as fractional pore area.

### Directed differentiation of EBs

Day 0 SA-EBs and FC-EBs were collected, washed with PBS, and incubated in Accutase for 10 min at 37 °C to dissociate. Accutase was quenched by adding 2X volume of E8 medium supplemented with 10 μM Y-27632, and cells were centrifuged at 200 g for 5 min. EB-dissociated cells were plated onto Matrigel-coated plates at a density of 65,000 cells/cm^2^ in E8 medium supplemented with 10 μM Y-27632 and allowed to attach overnight before beginning differentiation toward neuroectoderm or definitive endoderm. For neuroectoderm differentiation, cells were maintained in Essential 6 medium (Life Technologies) for 6 days. For definitive endoderm differentiation, cells were maintained in E8 medium for an additional 24 hrs prior to induction for 5 days (daily media change) with RPMI/B27 containing 100 ng/mL Activin A (R&D Systems).

### Flow cytometry

Briefly, hPSCs or EBs were collected, washed with PBS, and incubated with 0.25% trypsin/EDTA for 8–10 min at 37 °C followed by pipetting to dissociate. Trypsin activity was quenched by adding 2X volume of 20% FBS in RPMI supplemented with 5 μM Y-27632. Samples were centrifuged at 200 g for 5 min and pelleted samples were fixed with 1% paraformaldehyde for 20 min at room temperature, permeabilized with ice-cold 90% methanol for 15 min at 4 °C, and stored at −20 °C until processing. Samples were washed twice with Flow Buffer 1 (PBS containing 0.5% BSA) to remove residual methanol, incubated for 1 hr at room temperature with primary antibodies in Flow Buffer 2 (PBS containing 0.5% BSA and 0.1% Triton X-100), washed with Flow Buffer 2, and incubated in the dark for 30 min at room temperature with secondary antibodies in Flow Buffer 2. Samples were washed twice with FlowBuffer 2, resuspended in Flow Buffer 1, and stored on ice prior to data collection. Data were collected on a FACSCalibur flow cytometer and analyzed using FlowJo software. Positive expression was gated by <1% of the isotype control (Oct4 and Nanog) or against undifferentiated hPSCs (Pax6, Sox17, and FoxA2). Primary antibodies and dilutions used: mouse anti-human Oct3/4 (Santa Cruz Biotechnology, sc-5279, 1:60), rabbit anti-human Nanog (Cell Signaling Technology, 4903 S, 1:100), mouse anti-human Pax6 (Developmental Studies Hybridoma Bank, 1 ug/mL), mouse anti-human Sox17-AlexaFluor647 (BD Biosciences, 562594, 1:20), mouse anti-human FoxA2-PE (BD Biosciences, 561589, 1:20), normal mouse IgG_2b_ (Santa Cruz Biotechnology, sc-3879), and normal rabbit IgG (Santa Cruz Biotechnology, sc-3888). In assessments of Oct4 and Nanog expression in SA-EBs and FC-EBs, statistical significance between conditions was determined by two-tailed Student’s *t*-test (*p* < 0.05).

### Immunostaining of hPSCs on SAMs and EB sections

For immunostaining of hPSCs on SAMs, samples were fixed with 10% neutral buffered formalin for 15 min, permeabilized with 0.1% Triton X-100 in PBS for 5 min, blocked with 1% BSA in PBS for 30 min, and stained with primary antibodies (dilutions made in 1% BSA in PBS) for 1 hr at room temperature. Samples were washed three times with 0.05% Tween-20 in PBS and stained with secondary antibodies (dilutions made in PBS) for 1 hr at room temperature or overnight at 4 °C. Nuclei were counterstained with DAPI.

For histology and immunostaining of EB sections, EBs were collected from SAMs or agarose molds using wide-bore pipette tips, transferred to Eppendorf tubes, and allowed to settle before removing excess media and washing with PBS. EBs were then fixed with 10% neutral buffered formalin for 30 min at room temperature, washed twice with PBS, and incubated in 10% sucrose solution overnight at 4 °C before embedding in either Histogel for processing into paraffin blocks or O.C.T. compound at −80 °C for cryosectioning.

Paraffin-embedded EBs were sectioned at a thickness of 5 μm, deparaffinized in xylene, and stained with hematoxylin and eosin. Additional sections were assessed by immunohistochemistry for phosphorylated Smad3 (rabbit anti-human phosphoSMAD3 primary antibody; Thermo Scientific PA5–12693, 1:400). Briefly, tissue sections underwent antigen retrieval using 10 mM citrate buffer (pH 6.0) in an 80 °C water bath for 2 hours, followed by permeabilization with 0.25% Triton X-100. Sections were blocked with 10% BSA, and incubated in primary antibody for 1 hour at room temperature. Endogenous peroxidase activity was quenched and antibody detection was carried out using ImmPRESS anti-rabbit IgG HRP (Vector Labs). Finally, the signal visualized with ImmPACT DAB (Vector Labs) and sections counterstained with hematoxylin for contrast. Frozen samples were sectioned into 5–7 μm slices onto SuperFrost slides using a Leica CM1900 cryostat, and slides with cryosections were stored at −80 °C until further processing. For immunostaining of cryosectioned EBs, slides were equilibrated to room temperature for 15 min, fixed/permeabilized in ice-cold acetone for 10 min, and washed 3x with PBS before blocking with 10% BSA for 1 hr at RT. Slides were washed 3x with PBS before incubating overnight at 4 °C in mouse anti-human Oct3/4 (Santa Cruz Biotechnology, sc-5279, 1:100) and rabbit anti-human Nanog (Cell Signaling Technology, 4903 S, 1:100) primary antibodies, then washed 3x with PBS and incubated for 1 hr at room temperature in secondary antibody solution (goat anti-mouse Alexa Fluor488, goat anti-rabbit Alexa Fluor 568) containing DAPI. After overnight washes in PBS, stained sections were mounted in ProLong Gold Antifade Reagent, allowed to set overnight, and sealed with nail polish. Fluorescence was imaged with a Nikon Ti Eclipse microscope equipped with filters for FITC, Texas Red, and DAPI.

### Quantitative RT-PCR

For quantitative PCR (qPCR) analyses, EBs were washed with PBS and total RNA was isolated using the RNeasy mini kit (Qiagen) according to manufacturer’s instructions. RNA quality for all samples was assessed using the Agilent Bioanalyzer 3100. Only samples with RIN >7.0 were used for further analyses. RNA was reverse transcribed into cDNA using the RT^2^ First Strand Synthesis Kit (Qiagen). cDNA samples (4.5 ng input RNA/25 μL reaction for 96-well format RT^2^ Housekeeping Array, or 2.8 ng input RNA/10 μL reaction for 384-well format RT^2^ Custom Profiler Array) were mixed with RT^2^ Master Mix, loaded onto RT^2^ PCR Arrays (SABiosciences, Tables S1 and S2), and run on a LightCycler 4800 system according to the manufacturer’s protocol. Data analysis was performed using the ΔΔCt method, with *HSP90AB* as an internal control based on analysis of samples run on the RT^2^ Housekeeping Array (Supplementary Fig. S10). Ct values from the RT^2^ Housekeeping Array were analyzed using NormFinder software^68^ to identify a stable reference gene for the tested set of genes and samples. For the RT^2^ Custom Profiler Array, experimental conditions were run with at least three technical replicates per sample, with *n* = 3 independent biological replicates per condition.

### EB viability assessment

Day 0 EBs were collected in Eppendorf tubes and allowed to settle, and excess media was removed and replaced with staining solution. Cell viability was assessed using LIVE/DEAD staining kit (Life Technologies) according to manufacturer’s instructions and imaged immediately thereafter on a Nikon Ti Eclipse inverted epifluorescence microscope. For staining of intact EBs, CellTox Green (Promega) was used to visualize nuclei of EBs subjected to lysis following manufacturer’s instructions using 2X concentrated lysis buffer.

### Western blotting

Day 0 EBs were collected, washed with PBS, and resuspended in ice-cold RIPA buffer containing 1X Halt Protease/Phosphatase Inhibitor Cocktail. Samples were agitated for 15 min at 4 °C and spun at 12,000 g for 15 min at 4 °C. The supernatants from samples were collected and stored at −20 °C until use. Total protein was quantified by microBCA assay. Equal amounts of protein per sample were combined with Laemmli buffer, denatured for 5 min at 100 °C, loaded in 10% polyacrylamide gels and separated by SDS-PAGE. Proteins were transferred to PVDF membranes and incubated in blocking buffer (5% nonfat dry milk in TBST for 1 hr at RT. Membranes were incubated in primary antibodies in blocking buffer overnight at 4 °C, washed with TBST, and incubated in horseradish peroxidase (HRP)-conjugated goat anti-mouse or anti-rabbit IgG secondary antibodies in blocking buffer (Abcam, 1:10,000) for 1 hr at RT. Membranes were washed with TBST and incubated with ECL Western Blotting substrate (Pierce) for 1 min. Chemiluminescence was detected using a LAS4000 Mini imager and analyzed by densitometry using ImageJ’s gel plug-in. β-actin was used as a load control. Raw data were normalized such that SA-EB expression levels were adjusted to unity. Statistical significance between conditions was determined by two-tailed Student’s *t*-test (*p* < 0.05). Primary antibodies and dilutions used: rabbit anti-human phosphoSmad2/3 (Cell Signaling Technology, 3101 S, 1:1000), rabbit anti-human phosphoSmad1/5 (Cell Signaling Technology, 9516 S, 1:1000), and mouse anti-human β-actin (Abcam, ab8224, 1:1000).

### Statistical analysis

Unless otherwise stated, one-way analysis of variance (ANOVA) with Tukey’s method for multiple comparisons was performed to determine statistical significance (*p* < 0.05) between experimental groups.

### Data availability

The datasets generated and/or analyzed during the current study are available from the corresponding author on reasonable request.

## Electronic supplementary material

Supplementary Video 1

Supplementary Information
