# Supplementary material for: Controlled Self-assembly of Stem Cell Aggregates Instructs Pluripotency and Lineage Bias
Source: Sci Rep. 2017 Oct 25;7:14070. doi: 10.1038/s41598-017-14325-9 (PMC5656593; doi:10.1038/s41598-017-14325-9)
Supplement: Supplementary file 2 — Supplementary Information [file 41598_2017_14325_MOESM2_ESM.pdf]

Supplementary Information for the following manuscript:

Controlled Self-assembly of Stem Cell Aggregates Instructs Pluripotency and Lineage Bias

Angela W. Xie[a], Bernard Y.K. Binder[b], Andrew S. Khalil[a], Samantha K. Schmitt[c], Hunter J. Johnson[a], Nicholas A. Zacharias[a], and William L. Murphy[a,b,c,d,*]

[a]Department of Biomedical Engineering, University of Wisconsin-Madison, Madison, WI 53705

[b]Department of Surgery, University of Wisconsin-Madison, Madison, WI 53705

[c]Department of Materials Science and Engineering, University of Wisconsin-Madison, Madison, WI 53705

[d]Department of Orthopedics and Rehabilitation, University of Wisconsin-Madison, Madison, WI 53705

* Correspondence and requests for materials should be addressed to:
William L. Murphy
1550 Engineering Drive
Madison, WI 53706
(608) 262-2224
wlmurphy@wisc.edu

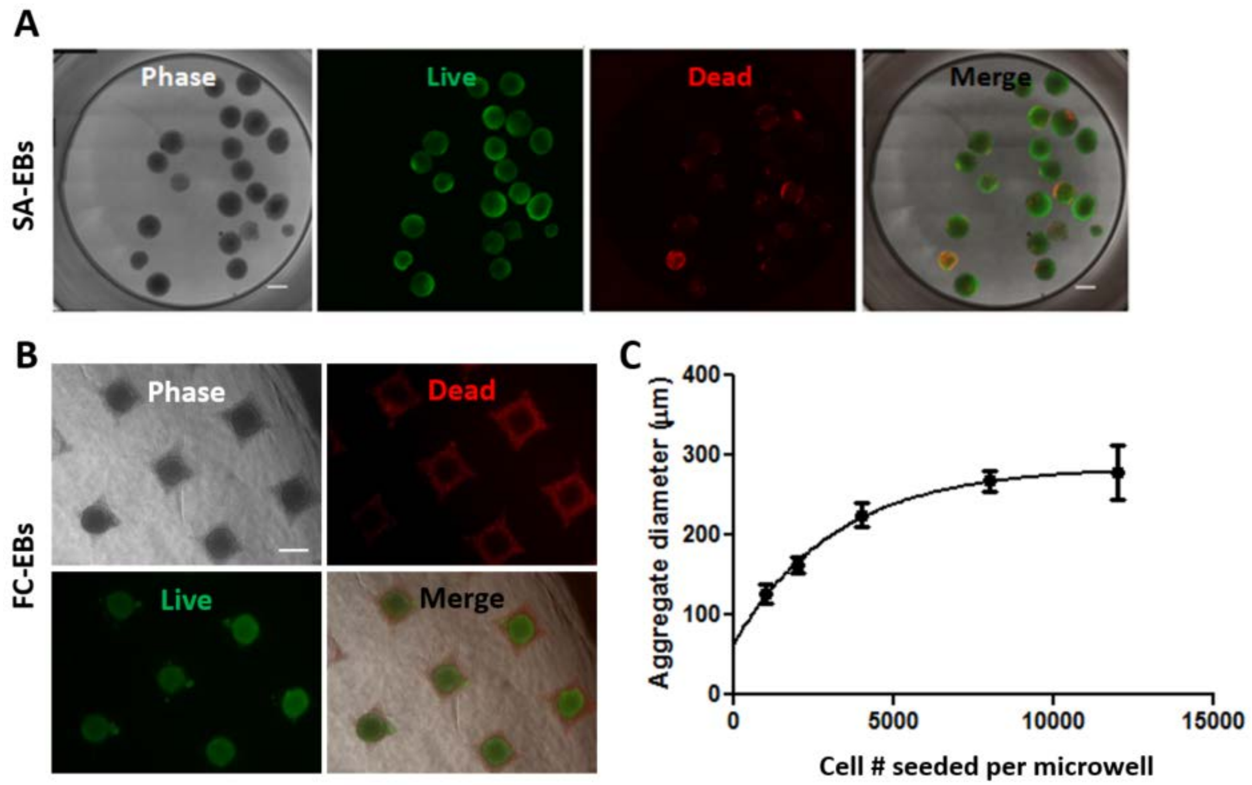

Supplementary Fig. S1. Characterization of viability and EB size in SA-EBs and FC-EBs. A) Viability of 5% cycRGDfC SA-EBs at day 0, assessed by LIVE/DEAD staining. Scale bar represents 500 µm. (B) LIVE/DEAD staining of FC-EBs at 24 hours after seeding. Not all cells within the microwells incorporated into EBs and those that did not incorporate were nonviable. (C) Control over FC-EB size. Varying initial cell numbers were centrifuged into agarose microwells and allowed to form EBs. EB diameter was assessed by microscopy at day 0.

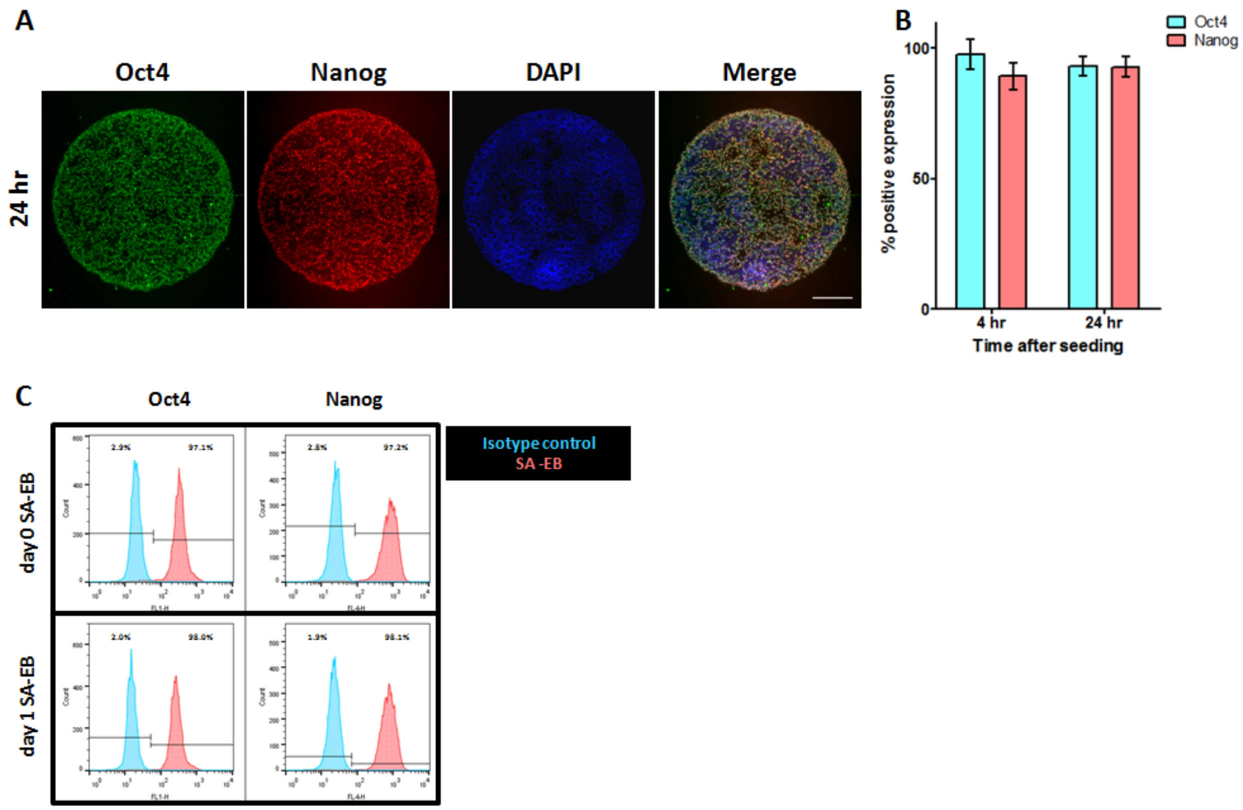

Supplementary Fig. S2. Assessment of Oct4 and Nanog expression in SA-EBs. (A) Immunofluorescence staining of hPSCs cultured on 5% cycRGDfC SAMs prior to self-assembly, at 24 hr after seeding. Cells were immunostained for Oct4 (green) and Nanog (red). DAPI was used to stain cell nuclei. Scale bar represents 250 μm. (B) Quantification of Oct4 and Nanog expression by hPSCs cultured on patterned 5% cycRGDfC SAMs at 4 hr and 24 hr after seeding. Immunofluorescence images at each time point were used to quantify percentage of positive cells per patterned spot. Error bars represent s.d. (C) Representative flow cytometry histograms quantifying expression of Oct4 and Nanog in day 0 and day 1 5% cycRGDfC SA-EBs. SA-EBs were formed and maintained in Essential 8 media, then collected and dissociated prior to staining with Oct4 and Nanog and evaluation by flow cytometry.

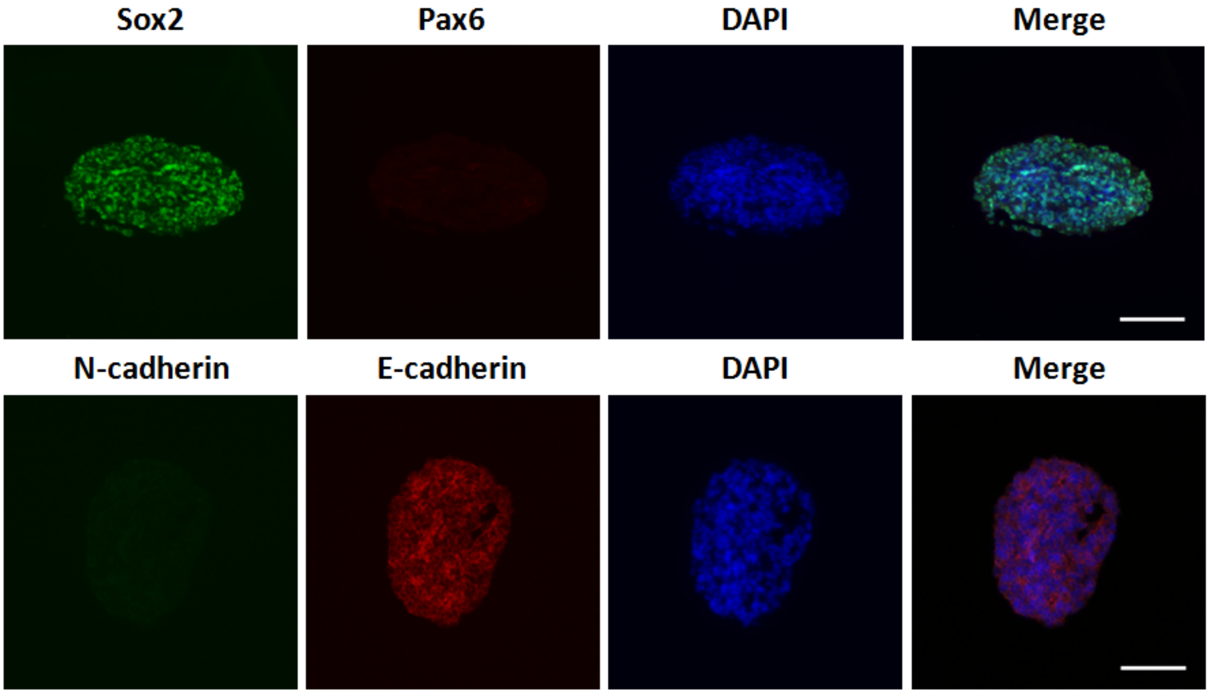

Supplementary Fig. S3. Immunofluorescence staining of day 0 SA-EBs for pluripotency and early differentiation markers. Cryosectioned EBs were stained for markers associated with pluripotency (Sox2, E-cadherin) or early differentiation (Pax6, N-cadherin). DAPI was used to stain nuclei. Scale bars represent 250 μm.

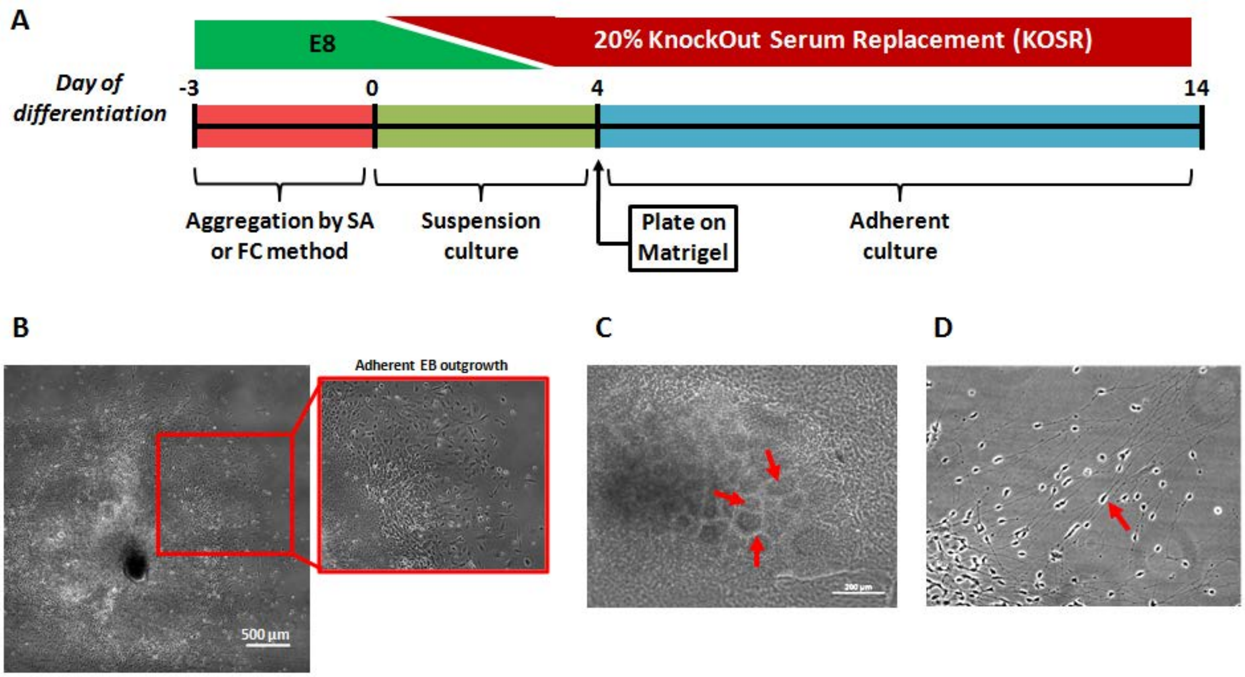

Supplementary Fig. S4. Spontaneous differentiation of EBs. (A) Schematic of protocol for spontaneous differentiation of SA-EBs and FC-EBs. EBs were transitioned from Essential 8 to differentiation medium ("DM" = 20% KOSR) between days 0 and 3 and maintained in DM until day 14. EBs were maintained in suspension culture (days 0-3) and plated on Matrigel at day 4 for further differentiation. (B) Plated EBs formed outgrowths containing differentiated cells. (C) Representative image of neural rosettes formed from plated FC-EBs at day 6. Rosettes were observed in >50% of FC-EB outgrowths. (D) Cells of neuronal morphology were found in day 9 FC-EB outgrowths.

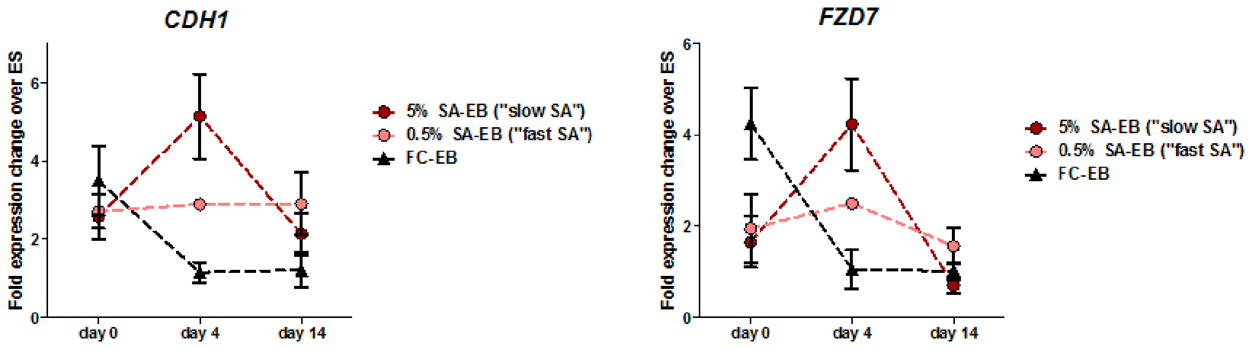

Supplementary Fig. S5. Self-assembly kinetics affects temporal expression of genes related to cell adhesion and Wnt signaling. *CDH1* (left) and *FZD7* (right) expression in slow and fast SA-EBs and FC-EBs at days 0, 4, and 14 during spontaneous EB differentiation. Fold-changes in expression are relative to undifferentiated hPSCs.

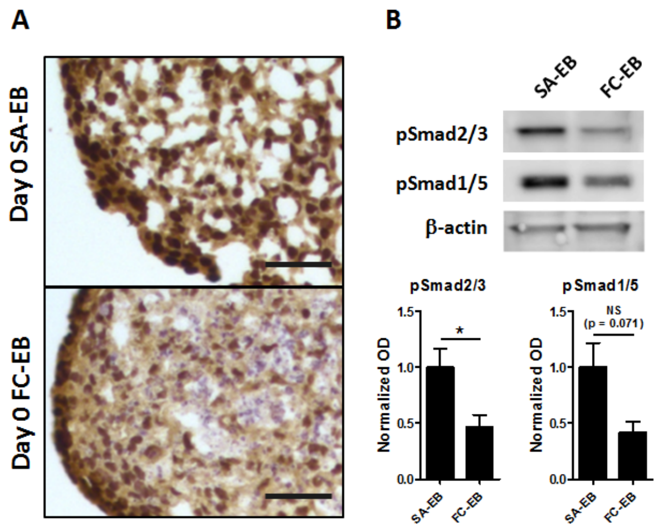

Supplementary Fig 6. Aggregation method influences TGFβ signaling in EBs. (A) Cryosections of day 0 SA-EBs and FC-EBs stained for phosphoSmad2/3. Scale bar represents 50 µm. (B) Western blot analysis of phosphoSmad2/3 and phosphoSmad1/5 expression in whole cell lysates from day 0 SA-EBs and FC-EBs. β-actin was used as a load control. Representative blots shown (top), with quantification by densitometry (bottom). Error bars represent s.e.m. from n = 4 independent biological replicates. * $p < 0.05$

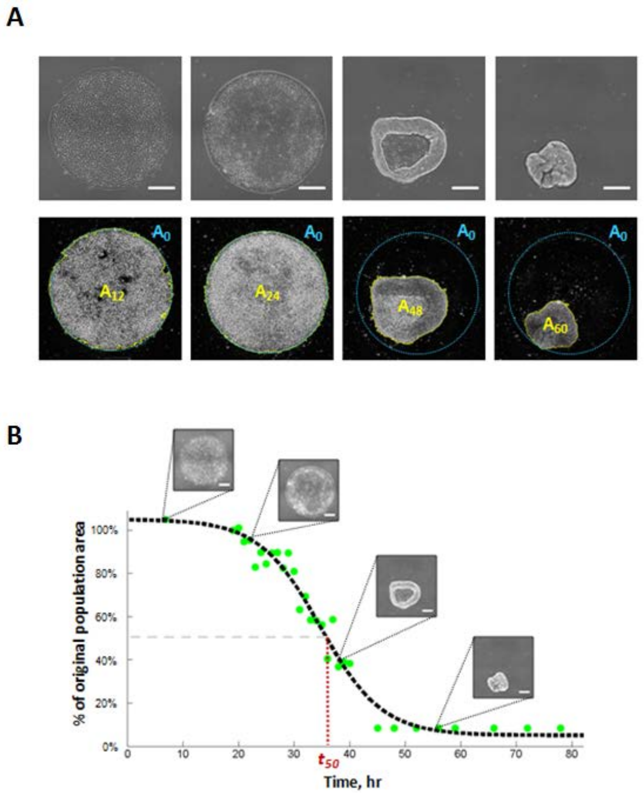

Supplementary Fig. S7. Method for quantification of SA-EB aggregation kinetics. (A) hPSCs were seeded onto patterned labile substrates and time lapse images of each patterned spot were acquired. Projected population area at each time point (A_n) was defined by edge detection and automated ROI drawing in NIS Elements analysis software. “Percent of original population area” was calculated as A_n normalized to initial area A_0 (defined as projected population area at 4 hrs after seeding), plotted as a function of time, and fit to a sigmoid curve. (B) Representative trace of EB self-assembly over time, with sigmoid fit showing determination of t_{50}.

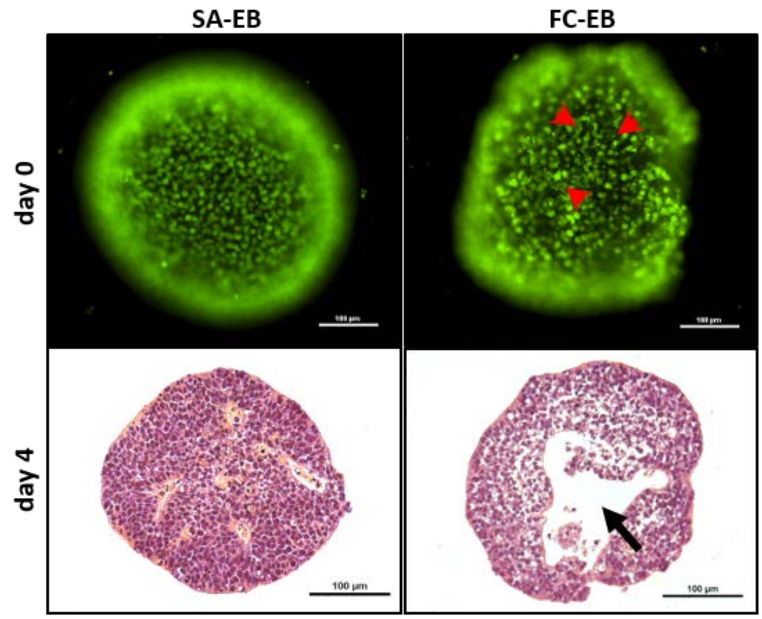

Supplementary Fig. S8. Aggregation method affects EB viability and necrotic core formation. (A) Optical sections of day 0 SA-EBs and FC-EBs, stained with CellTox Green DNA-binding dye. Red arrowheads denote fragmented nuclei indicative of poor cell viability. (B) H&E staining of paraffin-embedded day 4 (slow) SA-EBs and FC-EBs. EBs were maintained in suspension culture in Essential 8 media. Black arrow indicates presence of a necrotic core.

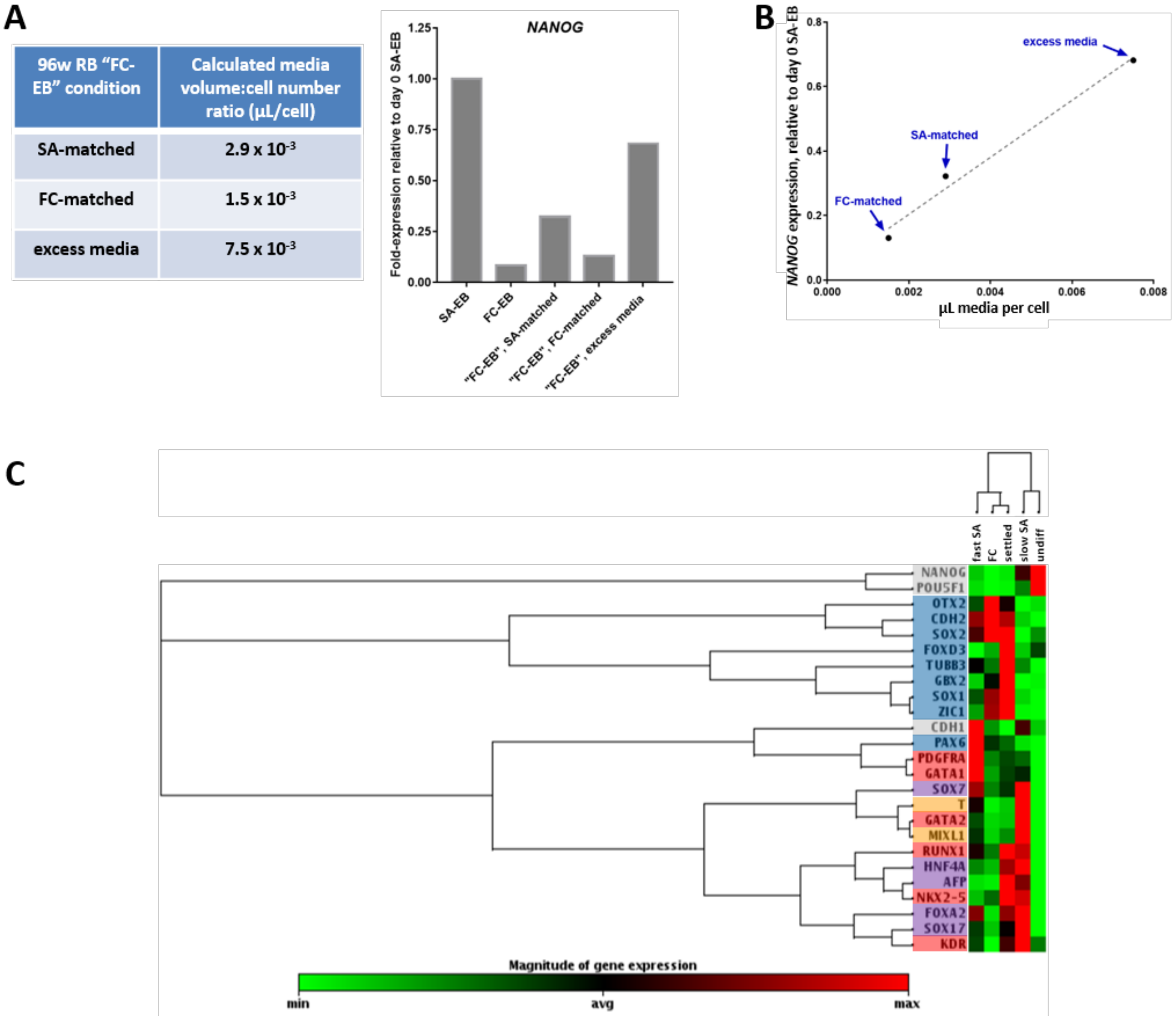

Supplementary Fig. S9. Aggregation method influences EB gene expression under equivalent culture conditions. (A) (left) Conditions for forming "FC-EBs" in 96-well low-adhesion roundbottom plates with media volume:cell number ratio matched to SA-EBs and FC-EBs. (right) *NANOG* expression in SA-EBs, FC-EBs, 96-well "FC-EBs" formed under SA- and FC-matched conditions and with a media volume:cell number ratio in excess of matched conditions. (B) *NANOG* expression in 96-well "FC-EBs" as a function of media volume per cell. (C) Non-supervised hierarchical clustering of day 14 pluripotency and differentiation gene expression for slow and fast SA-EBs, FC-EBs, settled EBs, and undifferentiated hPSCs. Colors denote association of genes with pluripotency (gray), ectoderm (blue), mesendoderm/primitive streak (orange), mesoderm (red), or endoderm (purple). Values represent the mean of n = 3 independent biological replicates. Settled EBs were formed under identical culture conditions as FC-EBs in agarose microwells, except without centrifugation.

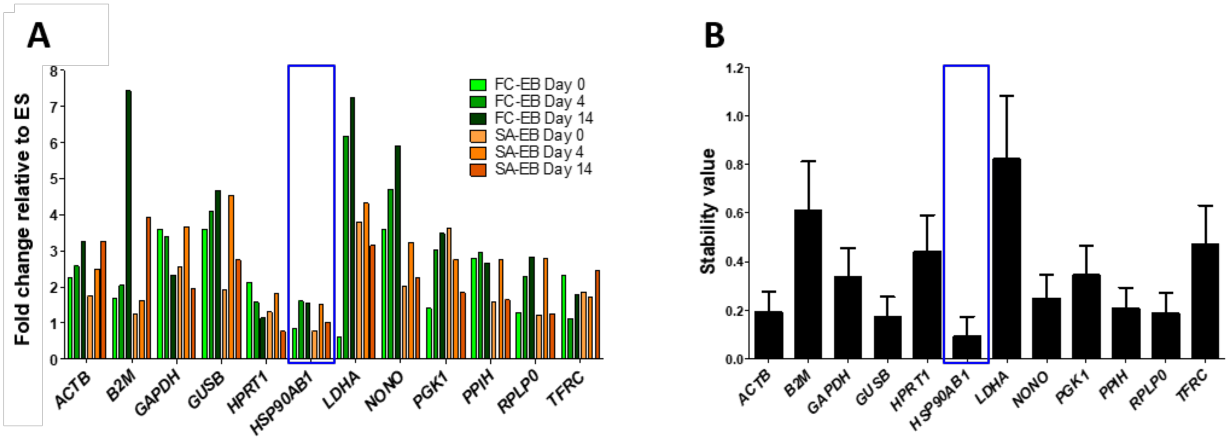

Supplementary Fig. S10. Identification of a stable reference gene in SA-EBs and FC-EBs throughout 14 days of spontaneous EB differentiation. (A) Fold-change expression of 12 reference genes in day 0, 4, and 14 SA and FC aggregates. qPCR data were analyzed by the ΔCt method. Fold-changes are expressed relative to undifferentiated hPSCs. Criteria for ideal reference genes included stable expression across both types of EBs as well as undifferentiated hPSC controls (i.e., fold-change ~ 1) and across time points. (B) Alternatively, Ct values from the same set of genes shown in (A) were analyzed using NormFinder software to identify a stable reference gene for the tested set of genes and samples. In this analysis, stable expression is indicated by a “stability value” closer to 0. *HSP90A* was identified as an appropriate reference gene via both approaches.

Supplementary Table S1. List of pluripotency and differentiation genes for RT[2] Custom Profiler PCR Array.

Gene Symbol	Refseq #	Description
AFP	NM_001134	Alpha-fetoprotein
CDH1	NM_004360	Cadherin 1, type 1, E-cadherin (epithelial)
CDH2	NM_001792	Cadherin 2, type 1, N-cadherin (neuronal)
FOXA2	NM_021784	Forkhead box A2
FOXD3	NM_012183	Forkhead box D3
FZD7	NM_003507	Frizzled family receptor 7
GATA1	NM_002049	GATA binding protein 1 (globin transcription factor 1)
GATA2	NM_032638	GATA binding protein 2
GBX2	NM_001485	Gastrulation brain homeobox 2
HNF4A	NM_178849	Hepatocyte nuclear factor 4, alpha
HSP90AB1	NM_007355	Heat shock protein 90kDa alpha (cytosolic), class B member 1
KDR	NM_002253	Kinase insert domain receptor (a type III receptor tyrosine kinase)
MIXL1	NM_031944	Mix paired-like homeobox
NANOG	NM_024865	Nanog homeobox
NKX2-5	NM_004387	NK2 homeobox 5
OTX2	NM_021728	Orthodenticle homeobox 2
PAX6	NM_000280	Paired box 6
PDGFRA	NM_006206	Platelet-derived growth factor receptor, alpha polypeptide
POU5F1	NM_002701	POU class 5 homeobox 1
RUNX1	NM_001754	Runt-related transcription factor 1
SOX1	NM_005986	SRY (sex determining region Y)-box 1
SOX2	NM_003106	SRY (sex determining region Y)-box 2
SOX7	NM_031439	SRY (sex determining region Y)-box 7
SOX17	NM_022454	SRY (sex determining region Y)-box 17
T	NM_003181	T, brachyury homolog (mouse)
TUBB3	NM_006086	Tubulin, beta 3
ZIC1	NM_003412	Zic family member 1

Supplementary Table S2. List of genes for RT^2 Housekeeping Array.

**UniGene**	**Refseq #**	**Gene Symbol**	**Description**
Hs.520640	NM_001101	*ACTB*	Actin, beta
Hs.534255	NM_004048	*B2M*	Beta-2-microglobulin
Hs.544577	NM_002046	*GAPDH*	Glyceraldehyde-3-phosphate dehydrogenase
Hs.255230	NM_000181	*GUSB*	Glucuronidase, beta
Hs.412707	NM_000194	*HPRT1*	Hypoxanthine phosphoribosyltransferase 1 (Lesch-Nyhan syndrome)
Hs.509736	NM_007355	*HSP90AB1*	Heat shock protein 90kDa alpha (cytosolic), class B member 1
Hs.2795	NM_005566	*LDHA*	Lactate dehydrogenase A
Hs.533282	NM_007363	*NONO*	Non-POU domain containing, octamer-binding
Hs.78771	NM_000291	*PGK1*	Phosphoglycerate kinase 1
Hs.256639	NM_006347	*PPIH*	Peptidylprolyl isomerase H (cyclophilin H)
Hs.546285	NM_001002	*RPLP0*	Ribosomal protein, large, P0
Hs.529618	NM_003234	*TFRC*	Transferrin receptor (p90, CD71)
